# Design, Synthesis, and Biological Evaluation of Anthracene Derivatives as Potent Antioxidants: Experimental, DFT, and Docking Studies

**DOI:** 10.1111/cbdd.70392

**Published:** 2026-09-06

**Authors:** Mohamed R. Elmorsy, Menna Moustafa, Hassan A. Etman, Nashwa M. El‐Metwaly, Safa A. Badawy

**Affiliations:** ^1^ Department of Chemistry, Faculty of Science Mansoura University Mansoura Egypt; ^2^ Department of Chemistry, College of Science Umm Al‐Qura University Makkah Saudi Arabia

**Keywords:** anthracene derivatives, antioxidant potential, DFT calculations, DPPH assay, KEAP1/Nrf2 complex, molecular docking, synthetic antioxidants

## Abstract

A series of 10 anthracene derivatives (**MM‐1‐MM‐10**), comprising anthracene cyanoacrylamides (**MM‐1‐MM‐5**) and anthracene hydrazinyl‐thiazoles (**MM‐6‐MM‐10**), was synthesized from anthracene‐9‐carbaldehyde and characterized by FT‐IR, ^1^H NMR, ^13^C NMR, mass spectrometry, and elemental analysis. The two‐family design was used to test whether replacing the cyanoacrylamide linker with a hydrogen‐donating hydrazinyl‐thiazole unit improves radical‐scavenging behavior while retaining the extended anthracene pi‐system. Antioxidant activity was evaluated by the DPPH assay using ascorbic acid as the reference. **MM‐10 and MM‐9** were the most active compounds, with IC_50_ values of 0.0033 ± 0.0002 and 0.0053 ± 0.0003 mg/mL, respectively, compared with 0.0164 ± 0.0006 mg/mL for ascorbic acid. MM‐7 and MM‐6 also showed strong activity, with IC_50_ values of 0.0123 ± 0.0006 and 0.0162 ± 0.0006 mg/mL, respectively. Gas‐phase DFT calculations were performed at the B3LYP/6‐311G(d, p) level. An exploratory descriptor‐activity analysis across the 10 compounds indicated that donor‐related descriptors, particularly the nucleophilicity index (Nu), were more closely associated with DPPH potency than the HOMO‐LUMO gap considered alone. Because the data set is small, this correlation is interpreted as hypothesis‐generating rather than as a generally predictive model. Docking against the KEAP1 Kelch domain (PDB ID: 8IVR) suggested favorable binding poses for **MM‐3**, **MM‐7**, **MM‐9**, and **MM‐10** relative to the co‐crystallized inhibitor. These docking results provide supportive mechanistic hypotheses at the pathway level but do not establish cellular KEAP1 inhibition or biological antioxidant activity. Overall, the experimental data identify the hydrazinyl‐thiazole subseries, particularly **MM‐7**, **MM‐9**, **and MM‐10**, as promising scaffolds for further cellular, toxicity, and in vivo evaluation.

## Graphical abstract

A series of anthracene‐based derivatives exhibited exceptional antioxidant activity, with IC_50_ values surpassing ascorbic acid. DFT and molecular docking studies revealed efficient charge transfer and strong Keap1/Nrf2 binding, highlighting their potential as promising therapeutic agents against oxidative stress.

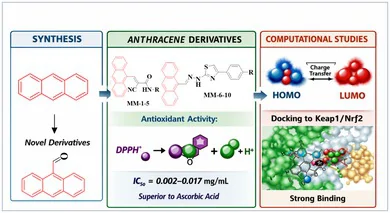

## 1 Introduction

Antioxidants are a recognized group of chemical compounds that are vital for normal blood flow and prevention of cardiovascular diseases, as they protect blood vessel membranes from damage (Gul et al. 2024). Antioxidants are chemicals that can delay or prevent oxidation (Ogunkunle et al. 2026). Synthetic antioxidants have recently gained popularity over their natural counterparts because of their accessibility and cost efficiency (Khan et al. 2023). Their primary role is to counterbalance free radicals produced during metabolic processes and mechanisms involved in protecting the gut from inflammation and injury (Wei et al. 2025; Keane et al. 2015). Free radicals are highly reactive molecules produced during redox reactions in cell metabolism. They are classified as reactive nitrogen, sulfur, or oxygen species (RSS, RNS, or ROS) because of their unpaired electrons. Free radicals, cofactors, and antioxidants are important for cellular signaling, gene expression, ion transport, age‐related diseases, and apoptosis (Khan et al. 2024). Oxidative stress occurs when reactive oxygen species (ROS) are produced at a faster rate than the biological system can neutralize or repair the damage they cause. (Adly 2010; Percival 1998; Mates et al. 1999). The inability of cells to neutralize reactive oxygen species (ROS) properly makes them a leading source of oxidative stress. As a result, biomolecules such as lipids, proteins, DNA, and RNA are damaged. Oxidative stress can impair all molecular targets, including DNA, proteins and lipids (Harman 1956). Cells possess an antioxidant defense system comprising endogenous and external elements that collaboratively destroy harmful radicals. This mechanism is designed to counteract oxidative damage (Nowacka et al. 2025; Becker 1993). Antioxidants are present on both sides of the mitochondrial membrane and help lower ROS‐induced stress. (Abou‐Hatab et al. 2017). Optimal antioxidants are redox metals that can operate in both aqueous and membrane environments, effectively scavenge free radicals at physiologically relevant concentrations, and be rapidly absorbed without interfering with the gene expression (Becker 1993). A significant group of organic molecules, known as polycyclic aromatic hydrocarbons (PAHs), are compounds with delocalized π‐electron systems and two or more fused benzene rings in their chemical structure (Tanious et al. 1992). Among them, the aromatic compound anthracene has three benzene rings that are fused in a linear fashion. Their conjugated π‐systems and extended aromaticity make them, research has demonstrated the intriguing photochemical and photophysical characteristics of anthracene derivatives (Becker and Norden 1999; Wilson et al. 1989). The biological effects of anthracenes on L1210 tumor cells in vitro have been well documented (Das et al. 2025). The anthracene nucleus can overlap with DNA base pairs because of its planar, linear, three‐ring structure (Kumar and Asuncion 1992). A variety of closely similar derivatives can be synthesized by taking advantage of the adaptable chemistry of the anthracene nucleus, which provides an efficient pathway for their production (Kumar et al. 1997). Spectroscopic monitoring of ligand binding to DNA is made possible by anthracene probes, which have high fluorescence quantum yields and modest absorption in the near‐UV region (Norden et al. 1992). The GC sequences of DNA cause the quenching of fluorescence from anthracene derivatives, whereas the AT sequences lead to an increase in anthryl fluorescence. This results in a valuable marker that can be used to determine the type of binding site (Narayanan et al. 2022). The triplet excited states of anthryl probes last for a long time, making them useful for inducing strand cleavage and causing substantial DNA damage. (Otake et al. 2023). Oxidative stress arises when the formation of reactive oxygen, nitrogen, or sulfur species exceeds the capacity of endogenous defense systems to neutralize them. Persistent oxidative imbalance can damage lipids, proteins, and nucleic acids and is associated with the progression of multiple inflammatory, metabolic, cardiovascular, and degenerative disorders (Narayanan et al. 2022). Small‐molecule antioxidants may reduce radical burden through hydrogen‐atom transfer (HAT), single‐electron transfer (SET), metal chelation, or combinations of these mechanisms. The DPPH assay is therefore widely used as an initial in chemico measure of radical‐scavenging capacity, although it does not by itself demonstrate cellular efficacy, target engagement, bioavailability, or therapeutic activity.

**FIGURE 1:**
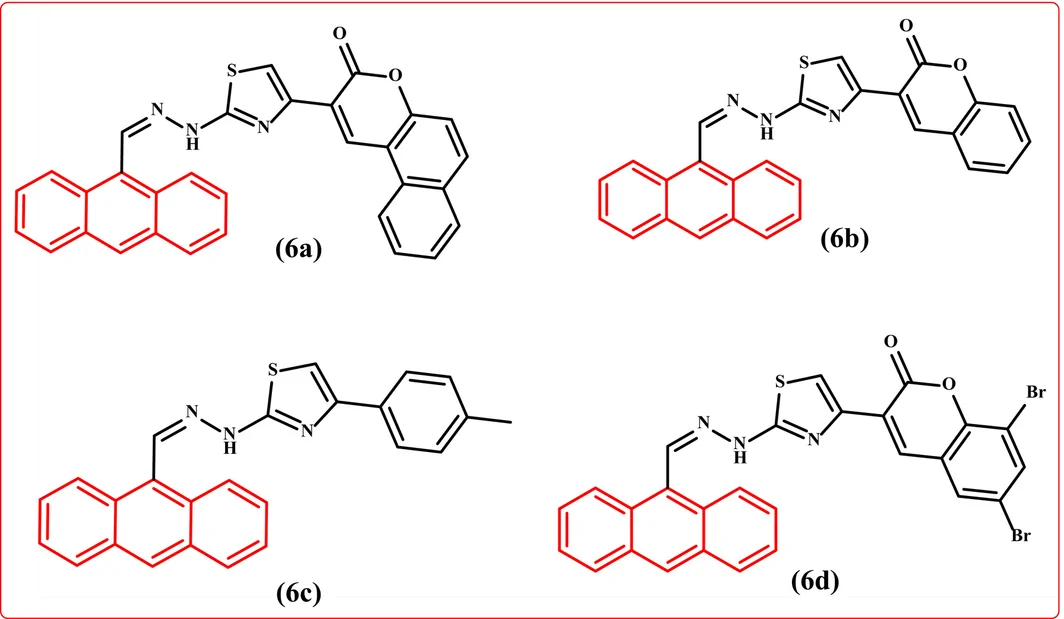
Biologically active compounds of anthracene‐9‐carbaldehyde 6a‐d (Hałdys et al. 2018).

Anthracene was selected as the central scaffold because its extended, rigid pi‐conjugated framework can delocalize electron density and stabilize radical intermediates. Functionalization at the 9‐position also provides a practical route for connecting electron‐donating or electron‐withdrawing groups without disrupting the fused aromatic core. Recent anthracene‐ and pyrene‐based Schiff bases have shown measurable DPPH‐scavenging activity and substituent‐dependent electronic behavior, supporting the use of polycyclic aromatic systems as tunable antioxidant platforms (Mostafa et al. 2024). Nevertheless, anthracene derivatives have been studied more extensively as fluorescent, DNA‐binding, and optoelectronic materials than as systematically optimized radical‐scavenging scaffolds. When the anthracene moiety is intercalated into the helix, the grooves are strategically occupied by substituents at positions 9 and 10 of the anthracene nucleus. (Mostafa et al. 2024). Anthracene derivatives have attracted considerable attention as privileged molecular scaffolds owing to their extended π‐conjugated architecture, rich electronic properties, and diverse biological activities. Their electron‐rich aromatic framework, together with the possibility of introducing electron‐donating or electron‐withdrawing substituents, provides an effective strategy for tuning redox behavior, charge‐transfer properties, and free‐radical‐scavenging efficiency, as shown in Figure 1 (Hałdys et al. 2018). Although anthracene derivatives have been widely studied as fluorescent probes, DNA‐binding agents, and anticancer compounds, their development as antioxidant scaffolds remains relatively limited (Otake et al. 2023). Therefore, the present compounds were designed to investigate how structural variations influence radical‐scavenging activity.

Compounds **MM‐1‐MM‐5** contain cyanoacrylamide units with different aryl, heteroaryl, carbazole, or bis‐anthracene substituents, which modify conjugation, steric effects, and donor–acceptor properties. In contrast, MM‐6–MM‐10 contain a hydrazinyl‐thiazole moiety with an accessible N–H bond and N/S heteroatoms that may stabilize charge and spin after hydrogen‐atom transfer or single‐electron transfer. Their substituents were selected to tune electron density, polarizability, and resonance delocalization. Accordingly, the hydrazinyl‐thiazole derivatives were expected to show stronger DPPH‐scavenging activity than the cyanoacrylamide derivatives. Experimental antioxidant evaluation was supported by quantum‐chemical calculations to examine electronic distribution, frontier molecular orbitals, charge‐transfer ability, and global reactivity descriptors. Molecular docking against the KEAP1 Kelch domain was also performed to explore possible interactions with the KEAP1–Nrf2 antioxidant pathway (Tomar et al. 2026; Mostafa et al. 2024). However, while the DPPH assay measures direct radical scavenging, docking only predicts potential binding; therefore, it is considered supportive rather than definitive evidence of KEAP1 inhibition.

The principal novelty of this study is the direct, internally controlled comparison of two structurally distinct anthracene‐derived families prepared from a common anthracene‐9‐carbaldehyde precursor: cyanoacrylamides (**MM‐1‐MM‐5**) and hydrazinyl‐thiazoles (**MM‐6‐MM‐10**). This design separates the contribution of the anthracene core from the effect of the linker and terminal substituent. The study further integrates quadruplicate DPPH measurements, an exploratory DFT descriptor‐activity analysis, and validated docking against the KEAP1 Kelch domain. The resulting family‐level trend—substantially stronger DPPH activity for the hydrazinyl‐thiazole derivatives—provides a specific scaffold‐design insight rather than merely reporting another set of substituted anthracenes.

## 2 Experimental Section

### 2.1 General Remarks

Detailed information on reagents, solvents, instrumentation, reaction monitoring, purification, and characterization is provided in the Supporting Information. FT‐IR, ^1^H NMR, ^13^C NMR, and mass spectra are supplied for representative compounds and, where available, for the complete series (Figures S1–S40). Peak assignments were made by correlating diagnostic functional‐group bands, proton integrations and multiplicities, carbon resonances, and molecular‐ion/fragment peaks with the proposed structures. Elemental‐analysis values are reported as measured and are interpreted together with the spectroscopic and mass‐spectral data. Close agreement between calculated and found values is not treated as independent proof of structure; small differences may arise from instrumental uncertainty, sample handling, residual solvent, or combustion efficiency.

### 2.2 Synthesis of Compounds MM‐1‐MM‐10

#### 2.2.1 General Method for the Synthesis of Compounds (MM‐1‐5)

Anthracene‐9‐carbaldehyde (2.06 g, 10 mmol) and the appropriate active‐methylene precursor 2‐cyano‐*N*‐(*p*‐tolyl)acetamide (**2**) (1.74 g, 10 mmol), 2‐cyano‐*N*‐(4‐methoxyphenyl)acetamide (**3**) (1.90 g, 10 mmol), 2‐cyano‐*N*′‐(2‐cyanoacetyl)acetohydrazide (**4**) (1.66 g, 10 mmol), 2‐cyano‐*N*‐(1*H*‐indol‐3‐yl)acetamide (**5**) (1.99 g, 10 mmol), and 2‐cyano‐*N*‐(9‐ethyl‐9*H*‐carbazol‐3‐yl)acetamide (Mostafa et al. 2024) (**6**) (2.77 g, 10 mmol) were dissolved in 100 mL dry ethanol, followed by the addition of piperidine (3–5 drops), a basic catalyst. The reaction mixture was heated under reflux for 3 h. were placed in a 50 mL round‐bottom flask and dissolved in dry ethanol (100 mL). Piperidine (3–5 drops) was added as the basic catalyst, and the reaction mixture was heated under reflux for 3 h. Reaction progress and consumption of the starting materials were monitored by thin‐layer chromatography under UV illumination. The product generally began to precipitate during reflux. At completion, the hot mixture was filtered, and the collected solid was washed thoroughly with cold ethanol and dried. The crude product was recrystallized from acetone/ethanol (1:1, v/v) to afford **MM‐1‐MM‐5** in isolated yields of 89%–94%. Each derivative was prepared in a separate reaction using the corresponding precursor.

#### 2.2.2 3‐(Anthracen‐9‐yl)‐2‐cyano‐N‐(p‐tolyl) acrylamide (MM‐1)

Yellow powder (92% yield), m.p. = 238°C–240°C. IR (KBr, cm^−1^): ν_max_ 3321 (N‐H), 3046 and 3012 (‐C=CH), 2917 (‐C‐H) aliphatic, 2219 (CN), 1683 (C=O).^1^H NMR (DMSO‐*d*_6_): *δ* 2.31 (s, 3H, CH_3_), 7.22 (d, *J* = 6.50 Hz, 2H, Ar‐H), 7.61–7.67 (m, 6H, Ar‐H), 8.15 (d, *J* = 10.00 Hz, 2H, Ar‐H), 8.20 (d, *J* = 6.50 Hz, 2H, Ar‐H), 8.80 (s,1H, Ar‐H), 9.19 (s, 1H, Ar‐H), 10.67 (s, 1H, N‐H) ppm. Mass analysis (m/z, %): 362 (M^⨥^, 68.41), 360 (46.23), 353 (41.27), 316 (40.90), 315 (100.00), 312 (42.06), 224 (44.51), 213 (40.25), 207 (91.01), 135 (47.52), 85 (43.91), 71 (93.28), 59 (44.84), 46 (50.49). Analysis for C_25_H_18_N_2_O (362.43): Calculated: 82.85; H, 5.01; N, 7.73%. Found: C, 82.9; H, 5.08; N, 7.82%.

#### 2.2.3 3‐(Anthracen‐9‐yl)‐2‐cyano‐N‐(4‐methoxyphenyl) acrylamide (MM‐2)

Yellow powder (94% yield); m.p. = 225°C–228°C. IR (KBr, cm^−1^): ν_max_ 3342 (N‐H), 3048 (‐C=CH), 2899 and 2832 (‐C‐H) aliphatic, 2216 (CN), 1678 (C=O). ^1^H NMR (DMSO‐*d*_6_): δ 3.35 (s, 3H, CH_3_), 7.00 (d, *J* = 10.00 Hz, 2H, Ar‐H), 7.61–7.70 (m, 6H, Ar‐H), 8.15 (d, *J* = 6.50 Hz, 2H, Ar‐H), 8.20 (d, *J* = 6.50 Hz, 2H, Ar‐H), 8.79 (s, 1H, Ar‐H), 9.18 (s,1H, Ar‐H), 10.63 (s, 1H, N‐H) ppm. ^13^C NMR (DMSO‐*d*_6_, ppm): δ 55.2, 113.7, 113.9, 115.0, 117.9, 121.8, 122.3 (2C), 125.1, 125.6, 125.9, 126.4, 126.6, 127.1, 128.2, 128.3, 128.7, 128.9, 129.4, 130.5, 130.6, 131.2, 150.1, 156.1, 159.1. Mass analysis (m/z, %): 378 (M, 70.23), 364 (67.42), 290 (54.16), 195 (75.67), 162 (100.00), 153 (67.10), 149 (57.85), 120 (71.98), 70 (62.23). Analysis for C_25_H_18_N_2_O_2_ (378.43): Calculated: C, 79.35; H, 4.79; N, 7.40%. Found: C, 79.41; H, 4.75; N, 7.38%.

#### 2.2.4 3‐(Anthracen‐9‐yl)‐N′‐(‐3‐(anthracen‐9‐yl)‐2‐cyanoacryloyl)‐2‐cyanoacrylohydrazide (MM‐3)

Red powder (90% yield), m.p. = 226°C–228°C. IR (KBr, cm^−1^): ν_max_ 3371 (N‐H), 3042 (‐C=CH), 2216 (CN), 1646 (C=O).^1^H NMR (DMSO‐*d*_6_): δ 7.60–7.63 (m, 4H, Ar‐H), 7.64–7.67 (m, 4H, Ar‐H), 8.02 (d, *J* = 10.00 Hz, 4H, Ar‐H), 8.20 (d, *J* = 10.00 Hz, 4H, Ar‐H), 8.77 (s, 2H, Ar‐H), 9.01 (s, 2H, Ar‐H), 10.87 (s, 2H, 2 N‐H) ppm. ^13^C NMR (DMSO‐*d6*, ppm): δ 115.0 (2C), 123.0 (2C), 124.4 (4C), 125.0 (4C), 125.3 (2C), 126.5 (4C), 126.8 (2C), 128.1 (4C), 128.5 (4C), 130.4 (4C), 147.2 (2C), 157.3 (2C) ppm. Mass analysis (m/z, %): 542 (M, 21.53), 511 (45.67), 458 (50.90), 448 (46.47), 441 (55.94), 423 (80.97), 404 (89.31), 384 (53.05), 352 (41.08), 310 (52.41), 305 (100.00), 249 (69.61), 217 (91.27), 193 (36.17), 139 (53.56), 94 (44.74), 71 (86.17). Analysis for C_36_H_22_N_4_O_2_ (542.60): Calculated: C, 79.69; H, 4.09; N, 10.33%. Found: C, 79.67; H, 4.17; N, 10.30%.

#### 2.2.5 3‐(Anthracen‐9‐yl)‐2‐cyano‐N‐(1H‐indol‐3‐yl) acrylamide (MM‐4)

Brown powder (90% yield), m.p. = 220°C–222°C. IR (KBr, cm^−1^): ν_max_ 3423–3342 (N‐H), 3049 and 3010 (‐C=CH), 2230 (CN), 1706 (C=O). ^1^H NMR (DMSO‐*d6*): δ 7.28–7.31 (m, 2H, Ar‐H), 7.35–7.42 (m, 4H, Ar‐H), 7.47 (d, 1H, *J* = 7.50 Hz, Ar‐H), 7.52 (d, *J* = 9.00 Hz, 1H, Ar‐H), 7.62 (t, *J* = 6.50 Hz, 1H, Ar‐H), 7.67 (t, *J* = 6.50 Hz, 1H, Ar‐H), 8.20 (d, 3H, *J* = 7.50 Hz, Ar‐H), 8.80 (s,1H, Ar‐H), 9.24 (s, 1H, Ar‐H), 10.79 (s, 1H, N‐H), 11.66 (s, 1H, N‐H) ppm. ^13^C NMR (DMSO‐*d6*, ppm): 103.4, 113.3, 114.2, 115.1, 1182, 118.6, 125.2, 125.9, 126.1, 126.5, 127.1, 127.5, 128.1, 128.3, 128.5, 128.7, 128.9, 129.7, 130.6, 132.4, 133.2, 134.3, 135.1, 130.0, 149.9, 159.2. Mass analysis (m/z, %): 387 (M, 20.18), 379 (32.44), 372 (49.69), 335 (61.34), 300 (60.06), 226 (49.56), 178 (87.30), 154 (51.81), 151 (46.80), 104 (59.35), 85 (100.00). Analysis for C_26_H_17_N_3_O (387.44): Calculated: C, 80.60; H, 4.42; N, 10.85%. Found: C, 80.67; H, 4.12; N, 10.91%.

#### 2.2.6 3‐(Anthracen‐9‐yl)‐2‐cyano‐N‐(9‐ethyl‐9H‐carbazol‐3‐yl) acrylamide (MM‐5)

Dark yellow powder (89% yield); mp. =230°C–232°C. IR (KBr, cm^−1^): ν_max_ 3405 (N‐H), 2926 and 2868 (‐C‐H), 2213 (CN), 1712 (C=O). ^1^H NMR (DMSO‐*d*_6_, ppm): *δ* 1.33 (t, *J* = 7.50 Hz, 3H, CH_3_), 4.45 (q, 2H, *J* = 7.50 Hz, CH_2_),7.22 (t, *J* = 7.50 Hz, 1H, Ar‐H), 7.48 (t, *J* = 7.50 Hz, 1H, Ar‐H), 7.61–7.64 (m, 3H, Ar‐H), 7.67–7.69 (m, 3H, Ar‐H), 7.80–7.82 (dd, *J* = 9.00, 2.00 Hz, 1H, Ar‐H), 8.14 (d, *J* = 6.50 Hz, 1H, Ar‐H), 8.21 (t, *J* = 10.00 Hz, 4H, Ar‐H), 8.56 (d, *J* = 7.50 Hz, 1H, Ar‐H), 8.81 (s, 1H, Ar‐H), 9.26 (s, 1H, Ar‐H), 10.83 (s, 1H, N‐H). Mass analysis (m/z, %): 465 (M, 30.85), 462 (43.12), 395 (29.15), 375 (63.81), 355 (78.15), 279 (53.28), 221 (35.24), 165 (44.02), 144 (28.47), 100 (100.00), 93 (42.75), 88 (45.19), 79 (48.68), 72 (64.81), 70 (75.61), 41 (54.50). Analysis for C_32_H_23_N_3_O (465.56): Calculated: C, 82.56; H, 4.98; N, 9.03%. Found: C, 82.52; H, 4.92; N, 9.09%.

#### 2.2.7 General Method for the Synthesis of Compounds (MM‐6‐10)

2‐(Anthracen‐9‐ylmethylene)hydrazine‐1‐carbothioamide (3.03 g, 10 mmol) and flask, 2‐(anthracen‐9‐ylmethylene)hydrazine‐1‐carbothioamide (3.03 g, 10 mmol) (**8**) (Hałdys et al. 2018), ethyl 2‐bromoacetate (**9**) (1.81 g, 10 mmol), 2‐chloro‐1‐(4‐fluorophenyl)ethan‐1‐one (**10**) (1.87 g, 10 mmol), 2‐oxo‐*N*‐(*p*‐tolyl) propane hydrazonoyl chloride (**11**) (2.28 g, 10 mmol), 2‐bromo‐1‐(4‐chlorophenyl)ethan‐1‐one (**12**) (2.53 g, 10 mmol) and 2‐bromo‐1‐phenylethan‐1‐one (**13**) (2.15 g, 10 mmol) were dissolved in 150 mL dry ethanol and drops of TEA as a basic catalyst the reaction mixture was heated under reflux for 3 h. The mixture was heated under reflux for 3 h. Reaction progress was monitored by TLC using petroleum ether/ethanol (9:1, v/v). The target product generally crystallized from the hot reaction medium. After completion, the mixture was filtered while hot, and the solid was washed with methanol and dried to afford **MM‐6‐MM‐10** in isolated yields of 85%–95%. Each derivative was prepared in a separate reaction using the corresponding electrophile.

#### 2.2.8 2‐(2‐(Anthracen‐9‐ylmethylene) hydrazinyl)‐5‐(5‐bromothiophen‐2‐yl) thiazole (MM‐6)

Olive powder (90% yield), m.p. = 260°C–262°C. IR (KBr, cm^−1^): ν_max_ 3382 (N‐H), 3110 and 3046 (‐C=CH), 1584 (C=*N*). ^1^H NMR (DMSO‐*d*_*6*_): *δ* 7.21 (d, *J* = 6.50 Hz, 1H, Ar‐H), 7.26 (s, 1H, Ar‐H), 7.34 (d, *J* = 6.50 Hz, 1H, Ar‐H), 7.56–7.58 (m, 2H, Ar‐H), 7.62–7.65 (m, 2H, Ar‐H), 8.15 (d, *J* = 6.50 Hz, 2H, Ar‐H), 8.65 (d, *J* = 6.50 Hz, 2H, Ar‐H), 8.69 (s, 1H, Ar‐H), 9.21 (s, 1H, Ar‐H), 12.58 (s, 1H, N‐H) ppm. ^13^C NMR (DMSO‐*d*_6_, ppm): *δ* 102.7, 110.5, 123.8, 124.6, 124.8, 125.6 (3C), 127.2 (2C), 129.1 (2C), 129.2, 129.3, 131.0 (2C), 131.4, 140.5, 140.6, 144.3, 144.8, 168.4 ppm. Mass analysis (m/z, %): 464 (M, 16.24), 393 (19.96), 380 (32.10), 312 (31.66), 225 (26.51), 217 (33.93), 174 (44.47), 141 (34.54), 139 (100.00), 128 (37.60), 112 (46.51), 110 (54.85), 101 (35.23), 66 (34.12), 44 (37.24). Analysis for C_22_H_14_BrN_3_S_2_ (464.40): Calculated: C, 56.90; H, 3.04; N, 9.05%. Found: C, 56.86; H, 2.99; N, 9.12%.

#### 2.2.9 2‐(2‐(Anthracen‐9‐ylmethylene) hydrazinyl)‐4‐(4‐fluorophenyl) thiazole (MM‐7)

Greenish‐yellow powder (85% yield); m.p. = 244°C–246°C. IR (KBr, cm^−1^): ν_max_ 3166 (N‐H), 3051 (‐C=CH), 1579 (C=*N*). ^1^H NMR (DMSO‐*d6*): δ 7.25 (t, *J* = 10.00 Hz, 2H, Ar‐H), 7.33 (s, 1H, Ar‐H), 7.56–7.59 (m, 2H, Ar‐H), 7.63–7.65 (m, 2H, Ar‐H), 7.91–7.93 (m, 2H, Ar‐H), 8.15 (d, *J* = 6.50 Hz, 2H, Ar‐H), 8.67 (s, 1H, Ar‐H), 8.68 (s, 2H, Ar‐H), 9.25 (s,1H, Ar‐H), 12.42 (s, 1H, N‐H) ppm. ^13^C NMR (DMSO‐*d*_6_, ppm): δ 103.4, 115.4, 115.6, 124.6 (2C), 125.0, 125.6 (2C), 127.1 (2C), 127.5, 127.6, 129.1 (4C), 129.3, 131.0 (2C),131.3, 141.5, 160.7, 162.6, 168.3. Mass analysis (m/z, %): 397 (M, 19.18), 362 (29.16), 358 (30.68), 341 (31.15), 265 (49.83), 263 (62.03), 183 (27.41), 90 (52.19), 89 (58.29), 73 (100.00), 72 (28.60). Analysis for C_24_H_16_FN_3_S (397.47): Calculated: C, 72.52; H, 4.06; N, 10.57%. Found: C, 72.59; H, 4.04; N, 10.63%.

#### 2.2.10 2‐(2‐(‐Anthracen‐9‐ylmethylene) hydrazinyl)‐5‐(‐(4‐chlorophenyl) diazenyl)‐4‐methylthiazole (MM‐8)

Dark red powder (95% yield), m.p. = 258°C–260°C. IR (KBr, cm^−1^): ν_max_ 3209 (N‐H), 3044 (C=CH), 2965 and 2917 (C‐H) aliphatic, 1590 (C=*N*). ^1^H NMR (DMSO‐*d*_6_): δ 2.63 (s, 3H, CH_3_), 7.14 (br.s, 2H, Ar‐H), 7.24 (br.s, 2H, Ar‐H), 7.60–7.62 (m, 2H, Ar‐H), 7.66–769 (m, 2H, Ar‐H), 8.18 (d, *J* = 10.00 Hz, 2H, Ar‐H), 8.81 (br.s, 3H, Ar‐H), 9.89 (s,1H, Ar‐H), 10.63 (s,1H, N‐H). ^13^C NMR (DMSO‐*d*_6_, ppm): δ 18.5, 114.3, 124.5, 125.0, 125.3, 125.6, 125.7, 125.6, 127.8 (4C), 129.1(3C), 129.7 (2C), 130.2, 130.5, 130.9, 131.2, 137.0, 141.0, 157.3, 158.9. Mass analysis (m/z, %): 457 (M + 2, 31.54), 455 (32.07), 440 (89.06), 424 (83.54), 407 (43.01), 383 (61.00), 361 (51.30), 351 (54.96), 326 (34.36), 287 (32.90), 269 (63.48), 253 (44.00), 247 (100.00), 236 (73.89), 204 (40.97), 180 (26.42), 120 (25.01), 95 (38.57), 86 (38.41). Analysis for C_25_H_18_ClN_5_S (455.96): Calculated: C, 65.85; H, 3.98; N, 15.36%. Found: C, 65.9; H, 4.05; N, 15.44%.

#### 2.2.11 2‐(2‐(Anthracen‐9‐ylmethylene) hydrazinyl)‐4‐(4‐chlorophenyl) Thiazole (MM‐9)

Deep yellow powder (85% yield); m.p. =260°C–262°C. IR (KBr, cm^−1^): ν_max_ 3164 (N‐H), 3048 (C=CH), 1580 (C=*N*). ^1^H NMR (DMSO‐*d*_6_, ppm): δ 7.41 (s, 1H, Ar‐H), 7.48 (d, *J* = 6.50 Hz, 2H, Ar‐H), 7.56–7.59 (m, 2H, Ar‐H), 7.62–7.65 (m, 2H, Ar‐H), 7.90 (d, *J* = 10.00 Hz, 2H, Ar‐H), 8.15 (d, *J* = 6.50 Hz, 2H, Ar‐H), 8.67 (s, 1H, Ar‐H), 8.68 (s, 2H, Ar‐H), 9.25 (s, 1H, Ar‐H), 12.43 (s, 1H, N‐H). ^13^C NMR (DMSO‐*d*_6_, ppm): δ 104.5, 124.6, 124.9, 125.5 (3C), 127.1 (3C), 127.3 (4C), 128.6 (3C), 129.0, 129.3, 131.0, 132.0,133.5, 140.2, 149.5, 168.3. Mass analysis (m/z, %): 413 (M, 27.20), 374 (80.13), 306 (35.54), 303 (45.46), 268 (36.42), 190 (43.15), 133 (74.07), 64 (82.31), 51 (39.83), 50 (100.00), 43 (45.86). Analysis for C_24_H_16_ClN_3_S (413.92): Calculated: C, 69.64; H, 3.90; N, 10.15%. Found: C, 69.69; H, 3.86; N, 10.13%.

#### 2.2.12 2‐(2‐(Anthracen‐9‐ylmethylene) hydrazinyl)‐4‐phenylthiazole (MM‐10)

Yellow powder (85% yield); m.p. = 240°C–242°C. IR (KBr, cm^−1^): ν_max_ 3166 (N‐H), 3124 and 3053 (‐C=CH), 1577 (C=*N*). ^1^H NMR (DMSO‐*d6*): δ 7.30–7.33 (m, 1H, Ar‐H), 7.35 (s, 1H, Ar‐H), 7.41–744 (m, 2H, Ar‐H), 7.57–7.59 (m, 2H, Ar‐H), 7.63–7.66 (m, 2H, Ar‐H), 7.89 (d, *J* = 10.00 Hz, 2H, Ar‐H), 8.15 (d, *J* = 10.00 Hz, 2H, Ar‐H), 8.67 (s, 1H, Ar‐H), 8.69 (s, 2H, Ar‐H), 9.25 (s,1H, Ar‐H), 12.42 (s, 1H, N‐H). ^13^C NMR (DMSO‐*d6*, ppm): δ 103.6, 124.6 (2C), 125.0, 125.5 (5C), 127.1 (2C), 127.6, 128.6 (4C),129.0 (2C), 129.3, 131.0 (2C), 134.6, 140.0, 168.1. Mass analysis (m/z, %): 379 (M, 18.73), 254 (32.33), 218 (76.99), 171 (39.37), 115 (45.92), 101 (41.48), 100 (36.91), 77 (100.00), 76 (90.52), 65 (80.05), 44 (65.67). Analysis for C_24_H_17_N_3_S (379.48): Calculated: C, 75.96; H, 4.52; N, 11.07%. Found: C, 75.03; H, 4.29; N, 10.19%.

### 2.3 DFT Calculations: Geometry Optimization and Global Reactivity Analysis

The initial geometries of **MM‐1‐MM‐10** were constructed and pre‐optimized in Chem3D 16.0 and saved in MOL format. Final geometry optimizations were performed in the gas phase with Gaussian 09 W using the B3LYP functional and the 6‐311G(d, p) basis set (Frisch 2009; Sarfraz et al. 2024). Harmonic frequency calculations were carried out at the same level of theory, and the absence of imaginary frequencies confirmed that the optimized structures corresponded to local minima. The optimized geometries were used to calculate frontier molecular orbitals, molecular electrostatic potential surfaces, and global reactivity descriptors. Cartesian coordinates of all optimized structures are provided in the Supporting Information. The selected level of theory provides a practical balance between computational cost and the description of the electronic structure of medium‐sized conjugated organic molecules and facilitates comparison across the complete series. No formal cross‐functional or basis‐set benchmark was performed; therefore, absolute orbital energies and derived descriptors are interpreted comparatively within this molecular set rather than as method‐independent observables.

### 2.4 DPPH Radical‐Scavenging Assay

The antioxidant activity of **MM‐1‐MM‐10** was evaluated using the DPPH radical‐scavenging assay, with ascorbic acid as the reference standard (Alanazi et al. 2025). Stock solutions were serially diluted in methanol. An equal volume of 0.135 mM DPPH solution was added to each concentration, and the mixtures were incubated for 30 min at room temperature in the dark. Absorbance was then measured at 517 nm against the corresponding blank.

(1) $$\begin{array}{c} \%{\text{DPPH}}^{•}\;\text{remaining}={\left[{\text{DPPH}}^{•}\right]}_{T}/\left[{\text{DPPH}}^{•}\right]T_{=0}\times 100 \end{array}$$

Each concentration was measured in four independent experiments (*n* = 4). Concentration‐response data were fitted with a four‐parameter logistic model in GraphPad Prism, and IC_50_ values are reported as mean ± SD (Alanazi et al. 2024). For comparisons with ascorbic acid, replicate‐based IC_50_ estimates were analyzed using two‐sided Welch's *t*‐tests followed by Holm correction for multiple comparisons. Adjusted *p* values below 0.05 were considered statistically significant. Lower IC_50_ values indicate stronger DPPH‐scavenging activity.

### 2.5 Molecular Docking Analysis

The X‐ray structure of the human KEAP1 Kelch domain in complex with the noncovalent inhibitor SIU (PDB ID: 8IVR; resolution 1.50 A) was obtained from the RCSB Protein Data Bank. Water molecules and non‐protein residues were removed, polar hydrogens were added, and Kollman charges were assigned using AutoDock Tools 1.5.7 (Goodsell et al. 2021). The co‐crystallized ligand was retained as the reference for defining the binding site and validating the docking protocol (KEAP1 2022). The three‐dimensional structures of MM‐1‐MM‐10 were generated and pre‐optimized with the MMFF94 force field. Polar hydrogens and Gasteiger charges were added, and all rotatable bonds were treated as flexible. Docking was performed with GNINA 1.3.2 using the co‐crystallized ligand to define the search space (autobox with 4 A padding), an exhaustiveness of 64, and nine retained poses per ligand (McNutt et al. 2025). The protocol reproduced the crystallographic pose of SIU with an RMSD of 0.23 A. Predicted affinities and CNN pose scores for MM‐1‐MM‐10 were compared with those of SIU. Because no experimental KEAP1 binding or Nrf2 activation assay was performed for the synthesized compounds, the docking results are interpreted only as supportive mechanistic hypotheses. The search space was centered on the co‐crystallized SIU ligand using GNINA's autobox function with 4 A padding. Docking was performed with an exhaustiveness of 64, and the nine top‐ranked poses were retained for rescoring with the GNINA convolutional‐neural‐network functions. Protocol validation, scoring, and visualization. Redocking of SIU reproduced the experimental pose with an RMSD of 0.23 A. The final **MM‐1‐MM‐10** poses were compared with SIU using classical docking affinity, CNN affinity, and CNN pose score, and interactions were visualized with Discovery Studio 2021. Cross‐docking against additional KEAP1 structures was not performed and is recognized as a limitation.

## 3 Results and Discussion

### 3.1 Synthesis and Structural Characterization of MM‐1‐5

**SCHEME 1:**
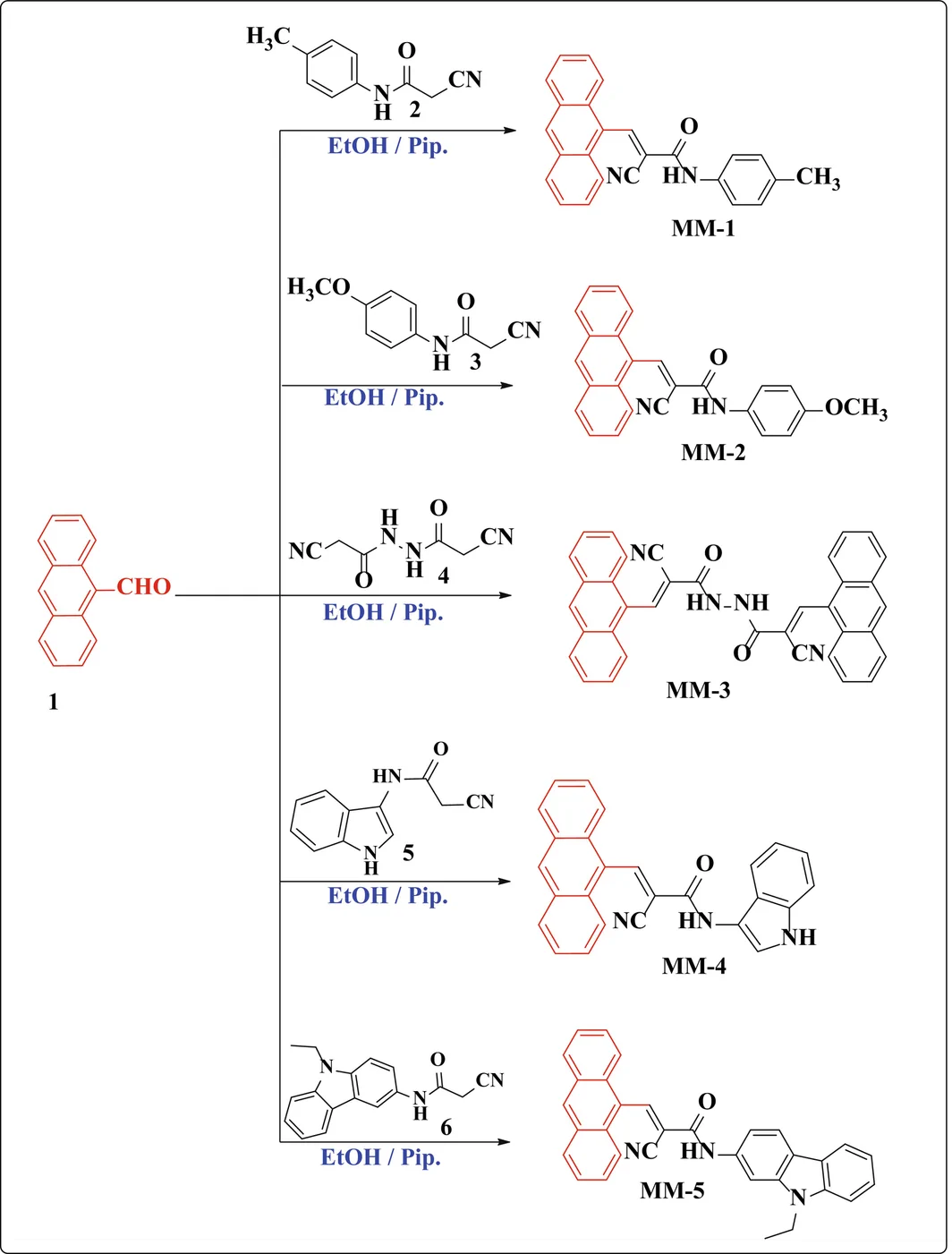
Synthesis of MM‐1‐5.

Scheme 1 presents a demonstration of the synthetic routes utilized in the manufacturing of organic molecules **MM‐1**‐**5**. Anthracene‐9‐carbaldehyde is combined with a wide variety of active methylene compounds derivatives (2–6) through the process of Knoevenagel condensation, resulting in excellent yields (85%–95%). These derivatives include 2‐cyano‐*N*‐(*p*‐tolyl) acetamide (Tomar et al. 2026), 2‐cyano‐N‐(4‐methoxyphenyl) acetamide (Tomar et al. 2026), 2‐cyano‐*N*′‐(2‐cyanoacetyl) acetohydrazide, 2‐cyano*‐N*‐(1*H*‐indol‐3‐yl) acetamide, and 2‐cyano‐*N*‐(9‐ethyl‐9*H*‐carbazol‐3‐yl) (Mostafa et al. 2024). The preferred molecules can be synthesized using Knoevenagel condensation. All target products were purified through recrystallization. The utilization of various analytical methods verified the presence of newly synthesized compounds. The infrared (IR) spectrum of **MM‐1** shows unique absorption bands related to the (N‐H) group at 3321 cm^−1^. The cyano (CN) group is observed at 2219 cm^−1^, and the carbonyl group (C=O) is observed at 1683 cm^−1^. A singlet signal at δ 2.31 ppm, which is related to the aliphatic protons (CH_3_), is revealed by the ^1^H NMR spectrum. On the other hand, the aromatic protons exhibited themselves as doublet, multiplet, and singlet signals throughout the range of δ 7.22–9.19 ppm, in addition to a singlet signal at δ 10.67 ppm that corresponds to the N‐H proton. Likewise, the molecular ion peak of **MM‐1**, which corresponds to the molecular formula (C_25_H_18_N_2_O), is shown by the mass analysis at m/z = 362 (68.41%). The infrared spectra of **MM‐2** demonstrate absorption peaks at 3342 cm^−1^ for the (N‐H) group and distinctive absorption peaks at 2899 and 2832 cm^−1^ for the (‐C‐H) aliphatic group. In addition, an absorption peak was observed at 1678 cm^−1^ for the (C=O) group. Two singlet signals at δ 3.35 and 10.63 ppm were detected in the ^1^H NMR spectrum, which are related to the aliphatic protons (CH_3_) and the NH group, respectively. The carbon atoms of the methoxy group and carbonyl of the amidic group resonated at δ 55.2 and 159.1 ppm, respectively, according to the ^13^C NMR spectra obtained. The organic compound **MM‐4** exhibited absorption bands in the infrared (IR) spectrum that were consistent with the presence of various functional groups. These included the following: two (NH) groups at 3423 and 3342 cm^−1^, a cyano (CN) group at 2230 cm^−1^, and a carbonyl (C=O) group at 1706 cm^−1^. The singlet signals of the two (NH) groups were observed at δ 10.79 and 11.66 ppm, respectively, in the ^1^H NMR spectra. In addition, a detectable signal was observed at δ 159.2 ppm in the ^13^C NMR spectrum of **MM‐4**, which is characteristic of the carbonyl group. The ^1^H NMR spectra of **MM‐5** revealed the presence of triplet and quartet signals at δ = 1.33 and 4.45 ppm, respectively, corresponding to the ethyl (CH_2_CH_3_) group. In addition, a singlet signal appeared for the (N‐H) group at δ 10.83 ppm. In addition, the mass analysis implies that the molecular ion peak of **MM‐5** matches the molecular formula (C_32_H_23_N_3_O) and has been detected at m/z = 465 (30.85%).

### 3.2 Synthesis of 2‐(anthracen‐9‐ylmethylene) hydrazine‐1‐carbothioamide (8)

**SCHEME 2:**
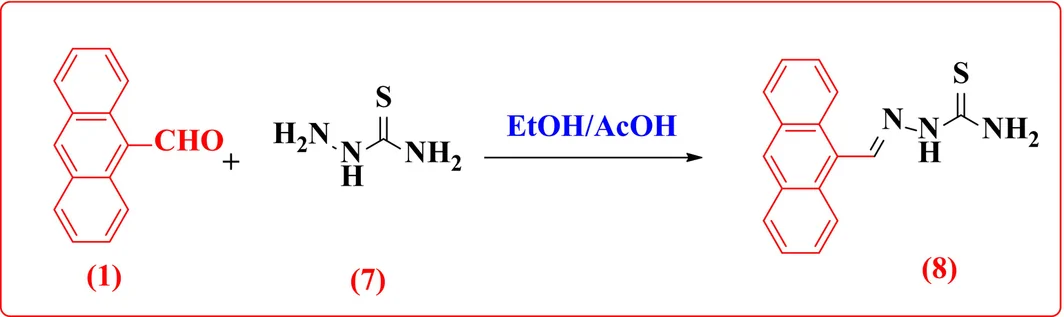
Synthesis of 2‐(anthracen‐9‐ylmethylene) hydrazine‐1‐carbothioamide (8).

As demonstrated in Scheme 2, a significant yield of compound (**8**) was achieved by combining anthracene‐9‐carbaldehyde (**1**) and hydrazinecarbothioamide (**7**) in 20 mL of dry ethanol with a catalytic quantity of glacial acetic acid (Hałdys et al. 2018). The reaction was carried out employing the Schiff base reaction.

**SCHEME 3:**
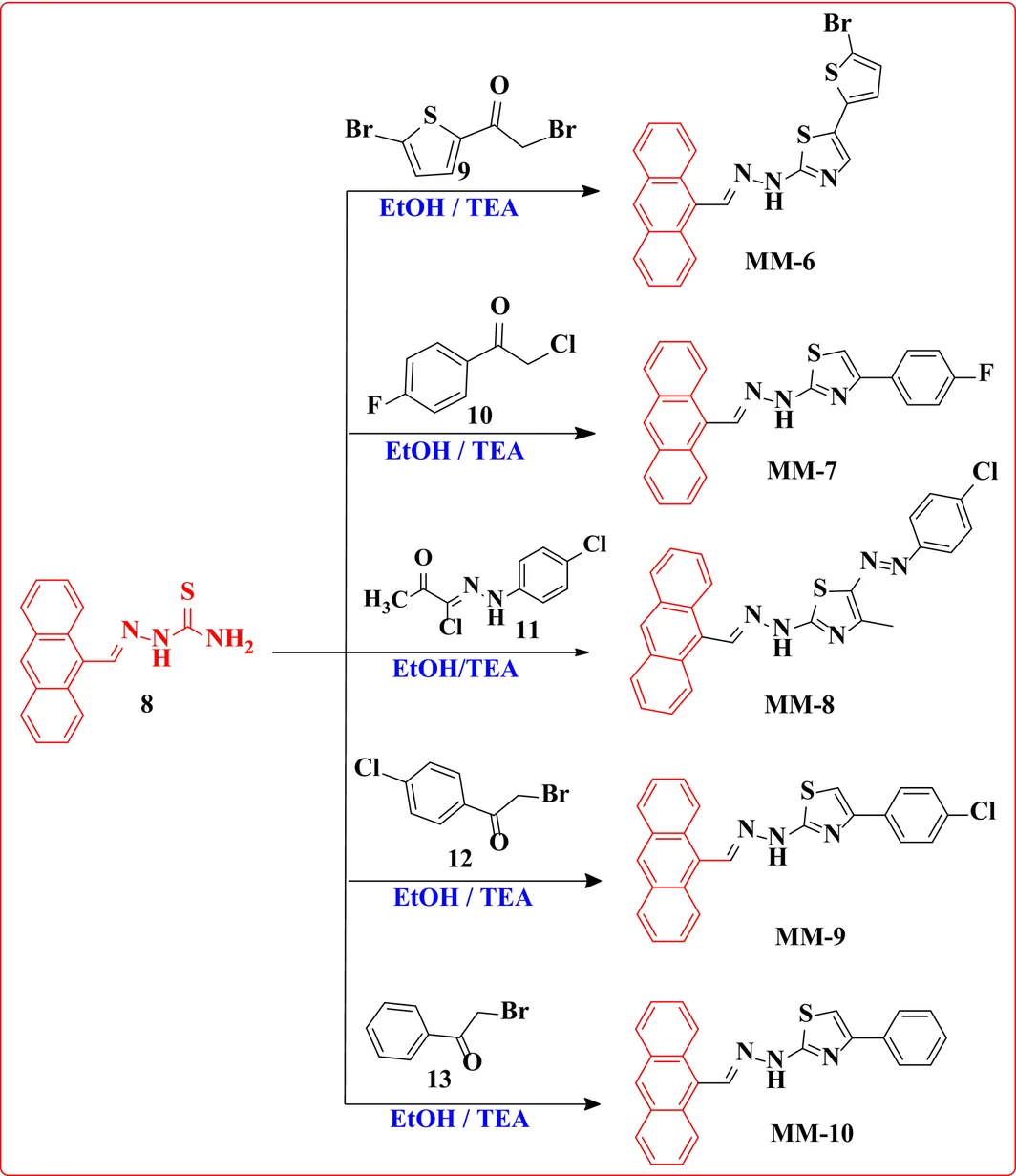
Synthesis of compounds **MM‐6‐MM‐10**.

In order to form a thiazole ring **MM‐6‐10**, the third scheme entails the cyclization of thiosemicarbazone (8) with a variety of halogenated compounds (**9–13**). The solid was separated by filtration once the mixture was complete, and then it was treated with methanol to continue the purification process. Elemental and spectroscopic tests confirmed the chemical structures of **MM‐6‐10**. In the infrared spectra of **MM‐6**, there are characteristic absorption bands that are recognized to the (N‐H) at 3382 cm^−1^, (‐C=CH) at 3110 and 3046 cm^−1^, and (C=*N*) at 1584 cm^−1^. The aromatic protons appeared as singlet, doublet, and multiplet signals, with a ranging from δ 7.21–9.21 ppm, in the ^1^H NMR. In contrast, the protons of (–NH) exhibited a singlet signal at δ 12.58 ppm. In addition, the ^13^C NMR spectra of **MM‐6** exhibit a detectable signal at δ 168.4 ppm, which is characteristic of (C=*N*). The ^1^H NMR spectra of **MM‐8** demonstrated two distinguishable singlet signals, with one of them being associated with the three aliphatic protons of (CH_3_) and the other related to the N‐H group at 2.63 and 10.63 ppm, respectively. The infrared (IR) spectrum of **MM‐9** shows unique absorption bands at 3164, 3048, and 1580 cm^−1^, which can be assigned to the functional groups (N‐H), (C=CH), and (C=*N*), respectively. Both **MM‐9** and **MM‐10** showed 24 carbon signals in their ^13^C NMR spectra. Compound **MM‐9** features a carbon atom (C=*N*) at δ 168.3 ppm, while compound **MM‐10** has a carbon atom (C=*N*) at δ 168.1 ppm (Scheme 3).

### 3.3 DFT Computational Studies of Anthracene Compounds

#### 3.3.1 Molecular Modeling of Compounds MM‐1‐10

**FIGURE 2:**
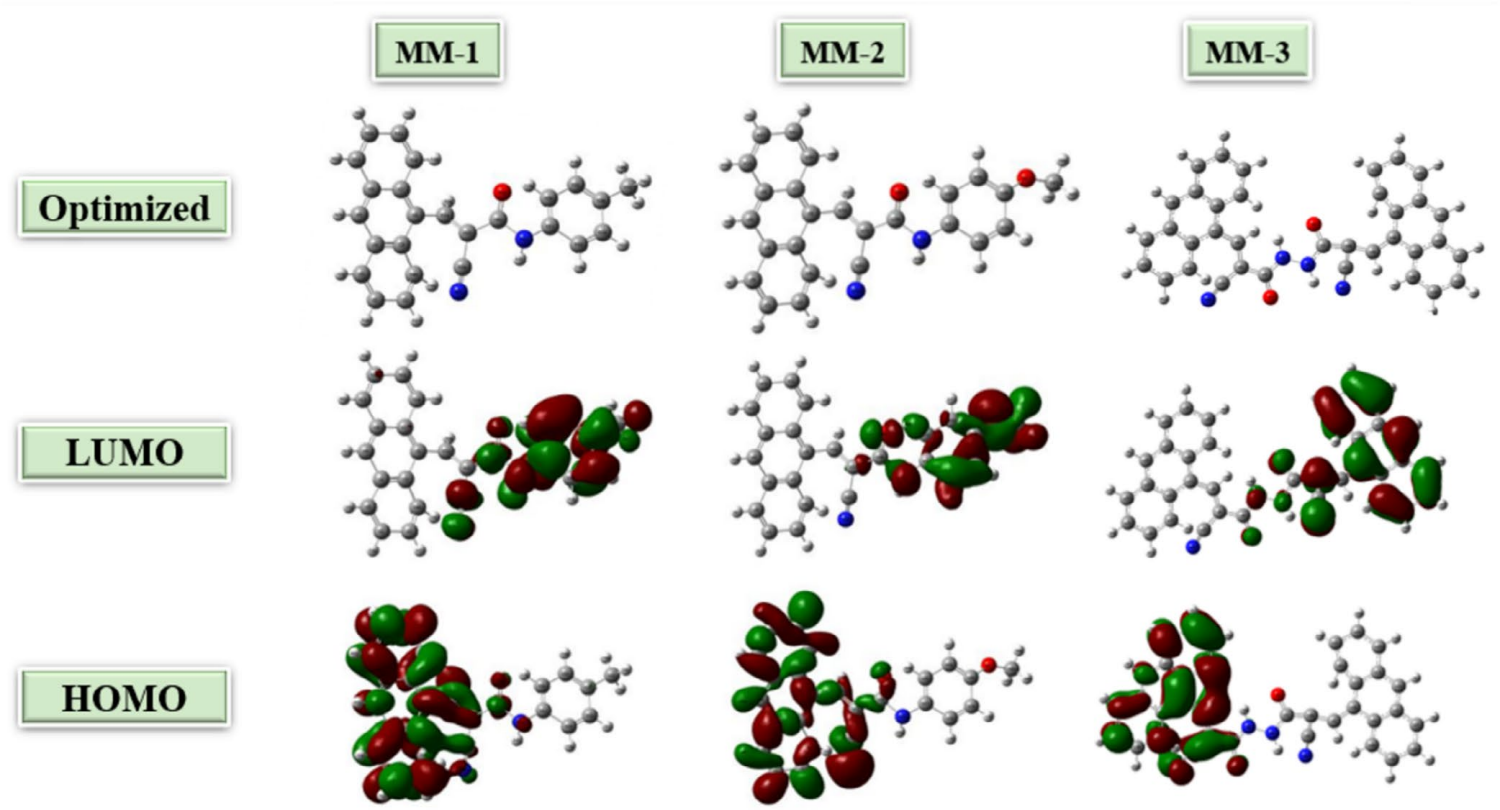
HOMO and LUMO profiles of compounds **MM‐1‐3**.

**FIGURE 3:**
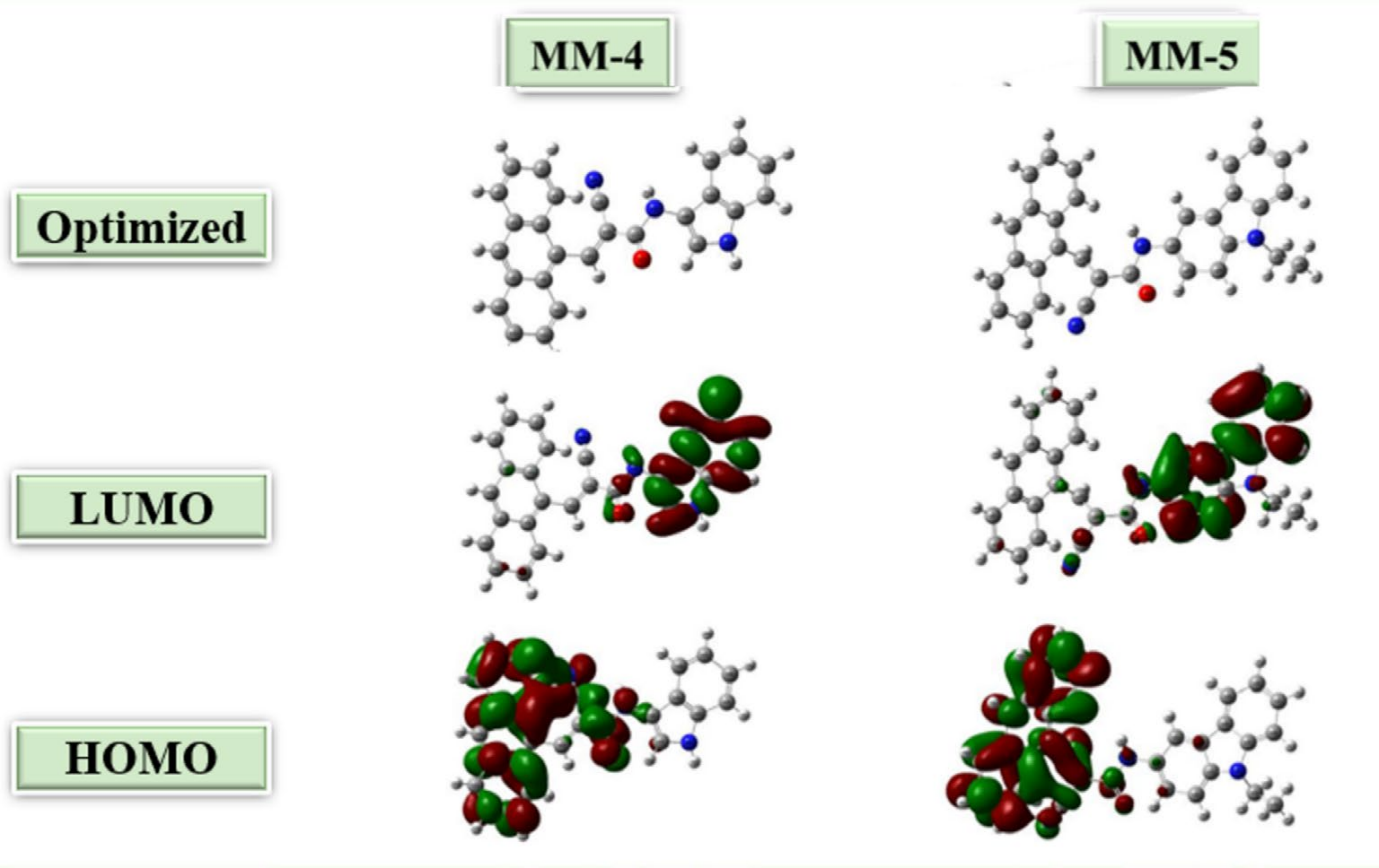
HOMO and LUMO profiles of **MM‐4** and **MM‐5**.

**FIGURE 4:**
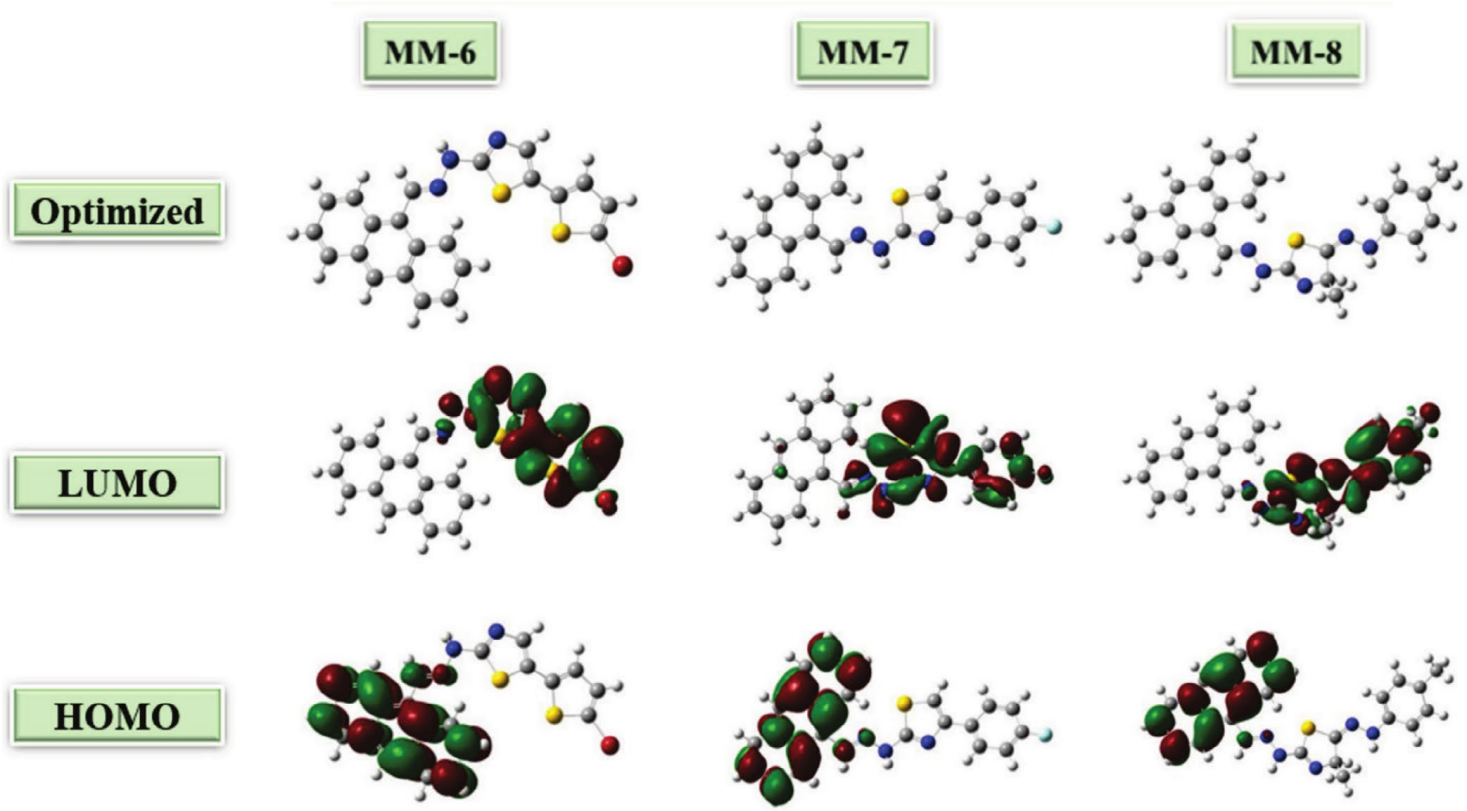
HOMO and LUMO profiles of compounds **MM‐6‐8**.

**FIGURE 5:**
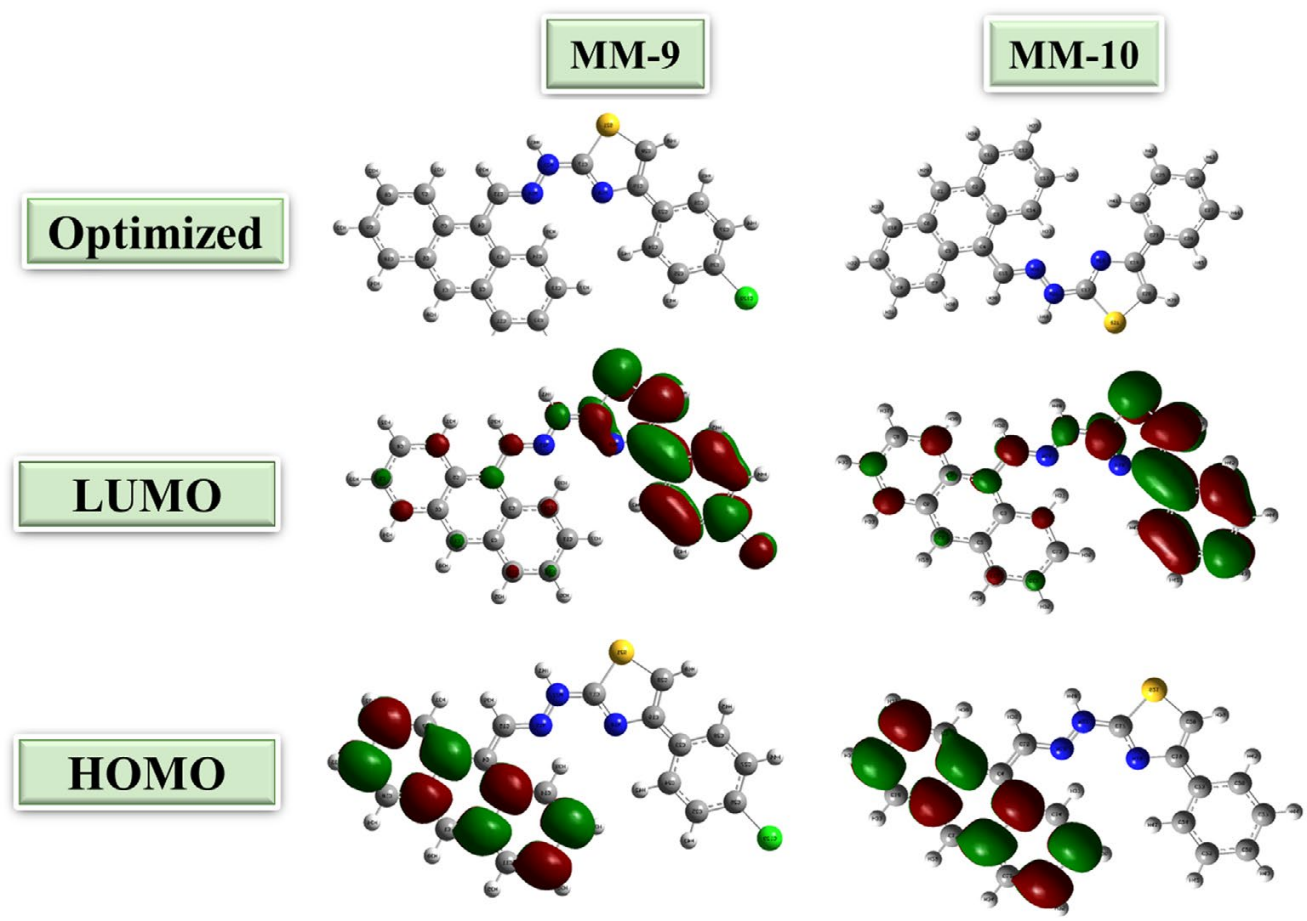
HOMO and LUMO profiles of **MM‐9‐10**.

Figures 2, 3, 4, 5 show the results of density‐functional theory (DFT) calculations performed on **MM‐1‐MM‐10** using the B3LYP hybrid method and the 6‐311G(d, p) basis set from the Gaussian 09 program (Moustafa et al. 2025; KEAP1 2022). The electron distributions of **MM‐1‐MM‐10** are presented in Figures 1, 2, 3, 4, which effectively illustrate their push‐pull characteristics. This figure shows that in the Highest Occupied Molecular Orbital (HOMO) for compounds **MM‐1‐MM‐10**, the electron density is predominantly concentrated on the donor segment (anthracene), whereas in the Lowest Unoccupied Molecular Orbital (LUMO), it is primarily localized on the acceptor segment. This distinct separation of electron density highlights the ability of the dyes to facilitate effective intramolecular charge transfer from the donor unit to the acceptor unit. The frontier molecular orbitals were estimated and explored. The HOMO and LUMO distributions indicate substituent‐dependent changes in electron density and intramolecular charge separation. These orbitals are useful for comparing electronic trends within the series; however, neither orbital localization nor the HOMO‐LUMO gap is treated as a direct measure of antioxidant activity. The computed descriptors are therefore evaluated in Table 1 together with the experimental DPPH data in the exploratory correlation analysis described below.

**TABLE 1:** Quantum chemical descriptors of the produced substances for the compounds **(MM‐1–MM‐10)**.

Compound	E_HOMO_	E_LUMO_	*E*_*g*_	*X*	*μ*	*ƞ*	*ω*	*Ʃ*	Nu
MM‐1	−5.41	−3.50	1.91	4.45	−4.45	0.95	10.42	0.52	0.09
MM‐2	−5.33	−3.53	1.80	4.43	−4.43	0.90	10.90	0.55	0.08
MM‐3	−4.37	−3.46	0.91	3.91	−3.91	0.45	16.98	1.11	0.05
MM‐4	−5.20	−3.39	1.81	4.29	4.29	0.90	10.16	0.55	0.09
MM‐5	−5.40	−3.42	1.98	4.41	−4.41	0.99	9.82	0.50	0.10
MM‐6	−5.62	−1.92	3.70	3.77	−3.77	1.85	3.84	0.27	0.26
MM‐7	−5.65	−1.94	3.71	3.79	−3.79	1.85	3.88	0.27	0.25
MM‐8	−5.22	−2.52	2.70	3.87	−3.87	1.35	3.72	0.37	0.26
MM‐9	−2.86	−0.05	2.81	1.45	−1.45	1.40	0.75	0.35	1.33
MM‐10	−2.75	−0.04	2.71	1.39	−1.39	1.35	0.71	0.37	1.40

*Note:* Where: *μ* = −*χ* = (E_HOMO_ + E_LUMO_)/2, *η* = (E_LUMO_ − E_HOMO_)/2, *σ* = 1/*η*, *ω* = μ^2^/(2*η*), Nu = 1/*ω*.

**FIGURE 6:**
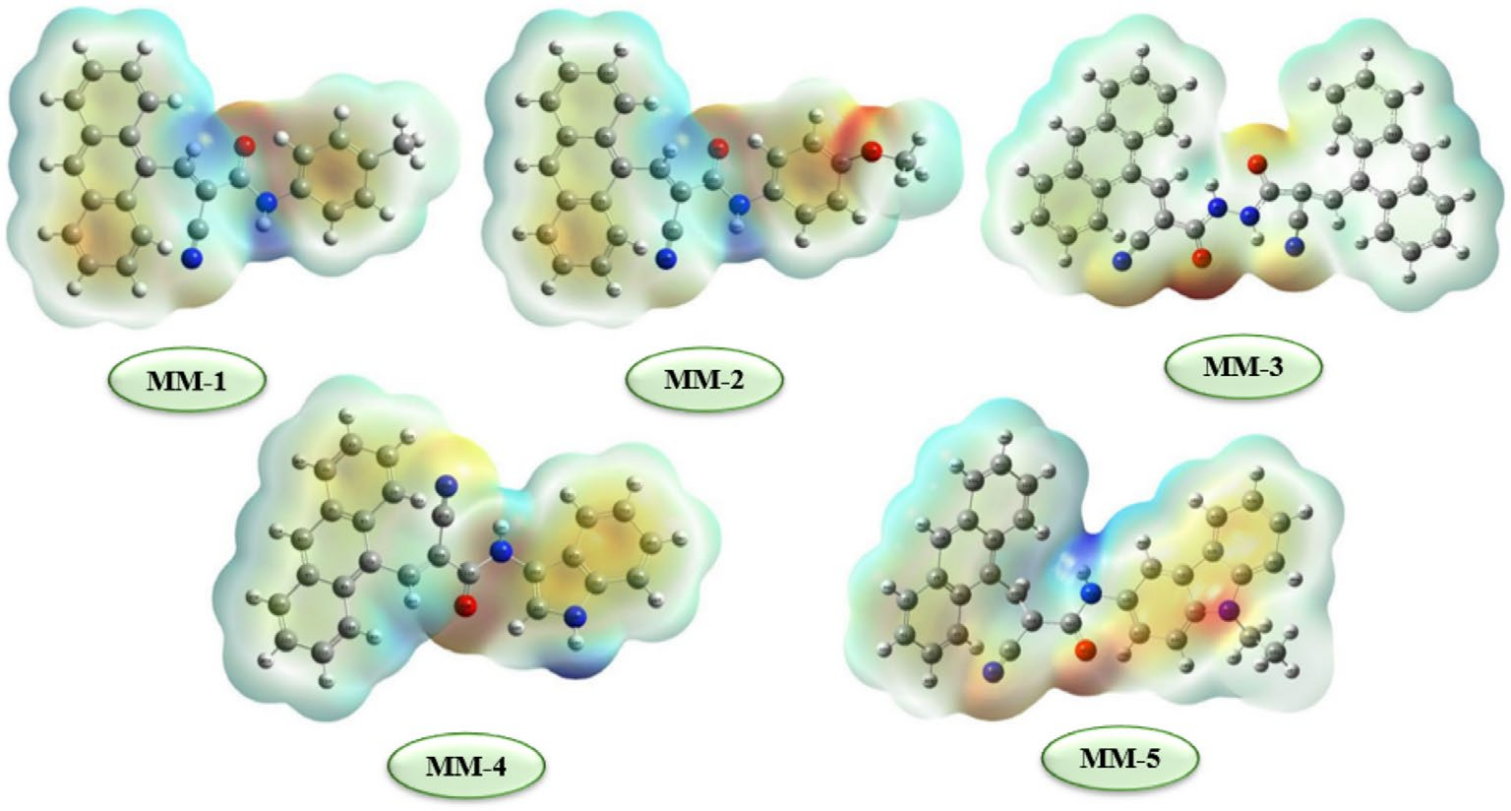
Molecular electrostatic potentials of **MM‐1**–**MM‐5**.

**FIGURE 7:**
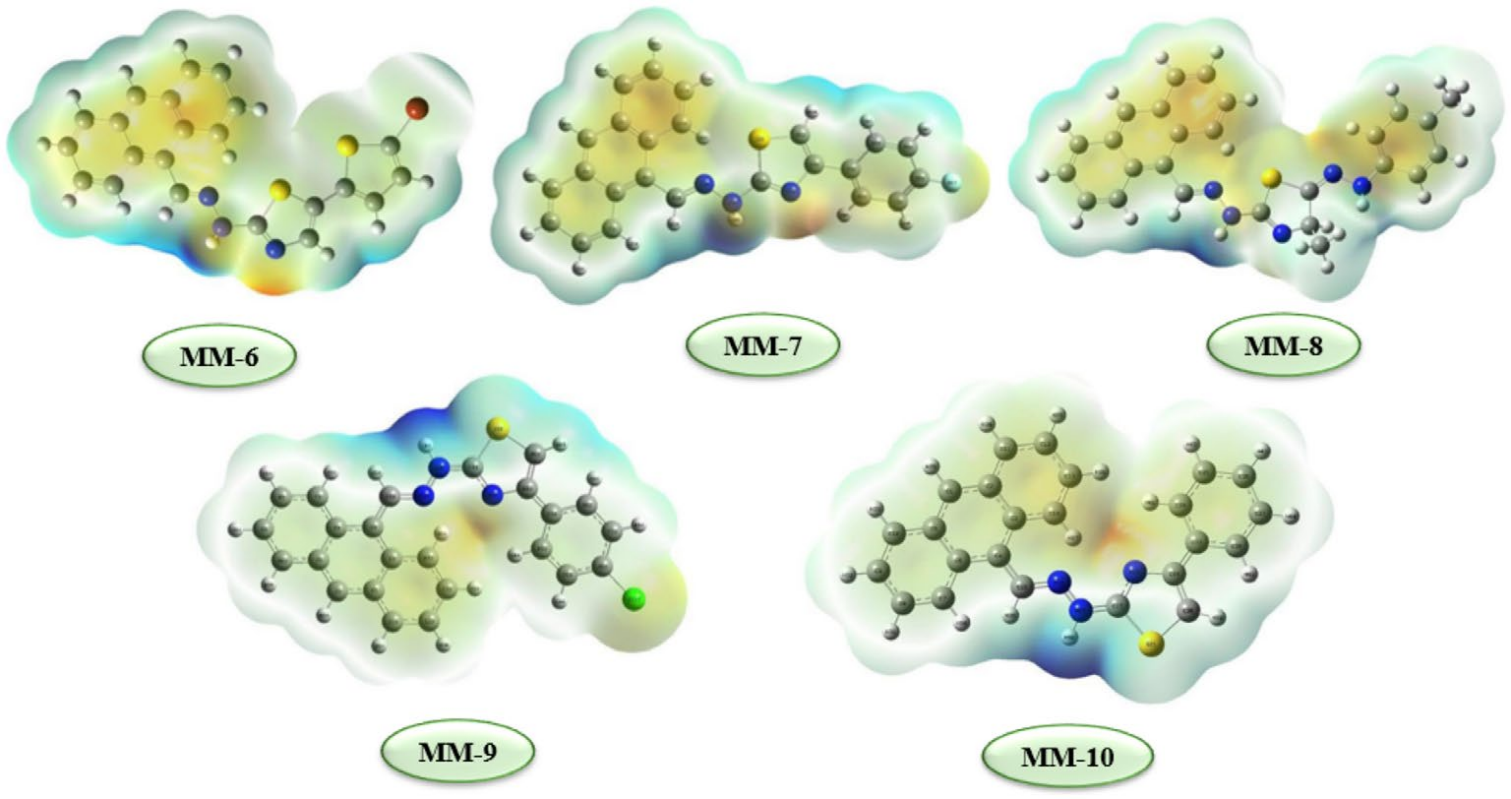
Molecular electrostatic potentials of **MM‐6**–**MM‐10**.

**FIGURE 8:**
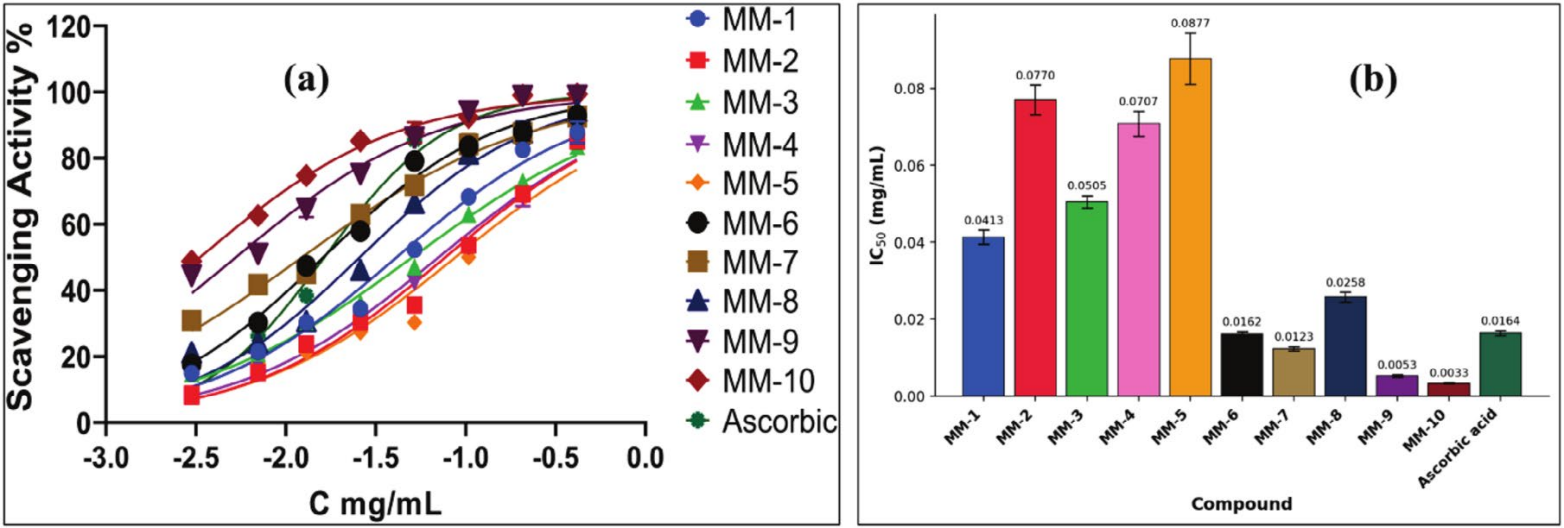
(a) Dose–response curves of compounds **MM‐1** to **MM‐10** and ascorbic acid (reference standard) against DPPH radicals. Each data point represents the mean ± SD of four independent replicates of the experiment. (b) Bar chart of the corresponding IC_50_ values (mg/mL, mean ± SD) of the synthesized derivatives compared with ascorbic acid, the reference standard.

**FIGURE 9:**
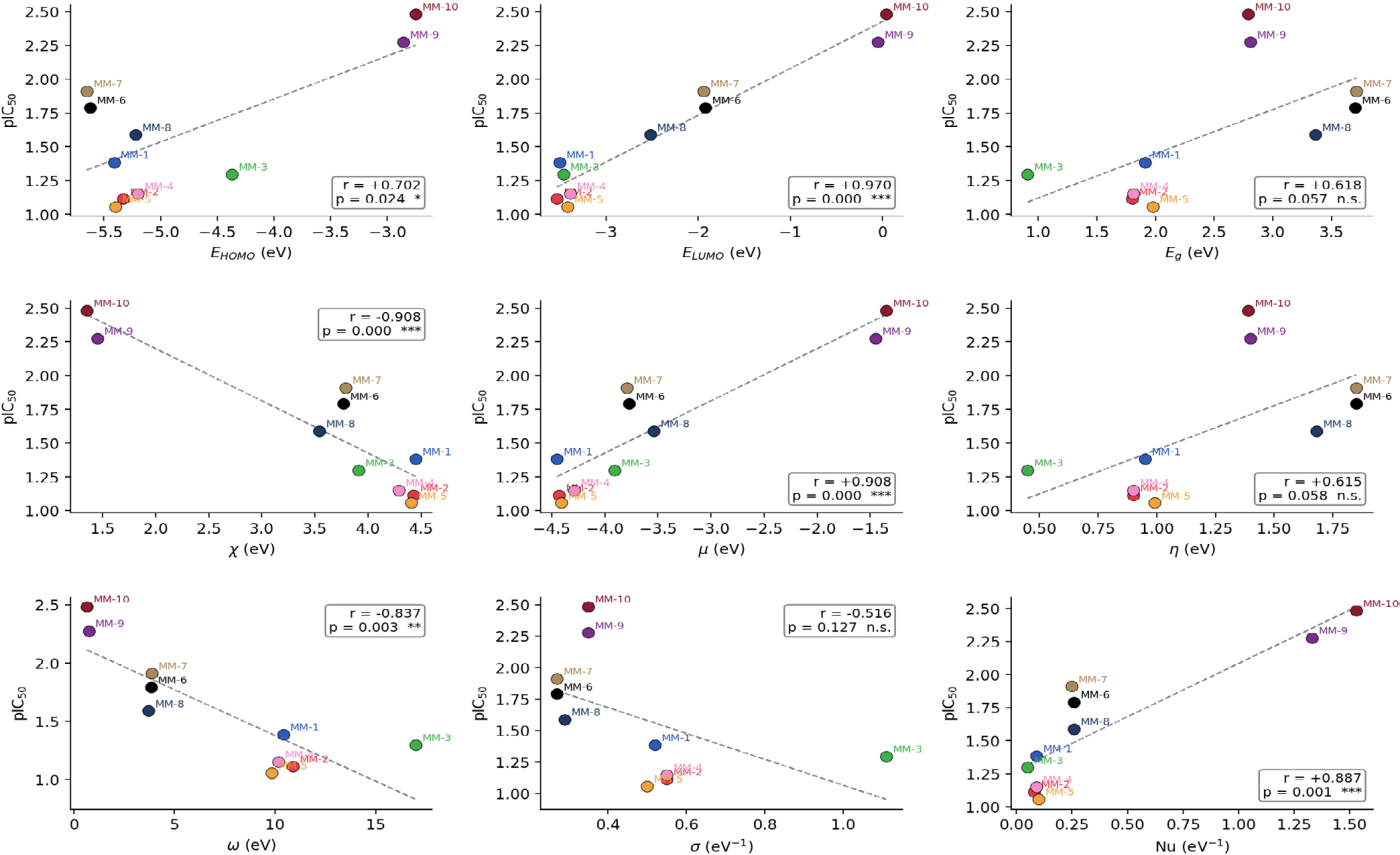
Complete scatter of the experimental pIC_50_ values versus the nine DFT‐derived quantum chemical descriptors listed in Table 1 (E_HOMO_, E_LUMO_, E_g_, *χ*, *μ*, *η*, *ω*, *σ*, Nu). Each point represents a compound (**MM‐1** to **MM‐10**).

**FIGURE 10:**
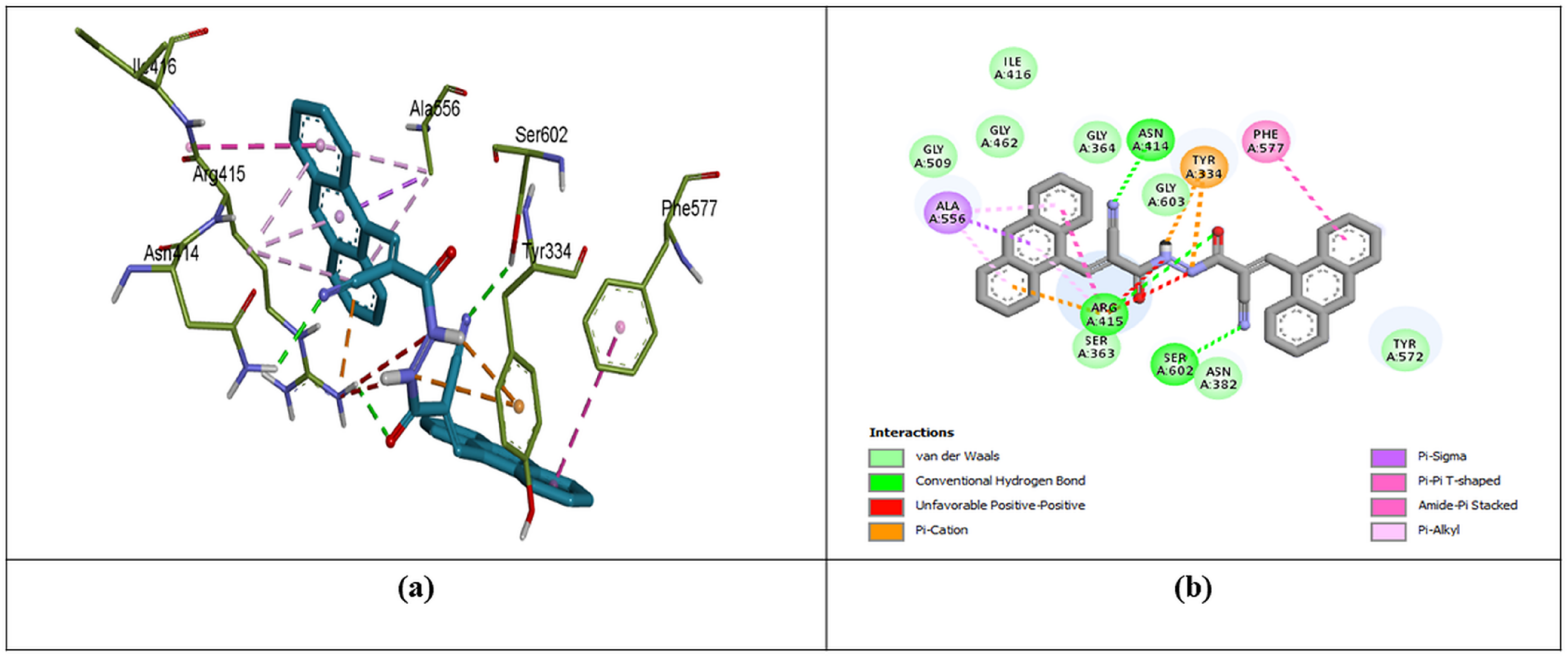
Predicted binding pose and two‐dimensional interaction map of **MM‐3** in the KEAP1 Kelch‐domain pocket.

**FIGURE 11:**
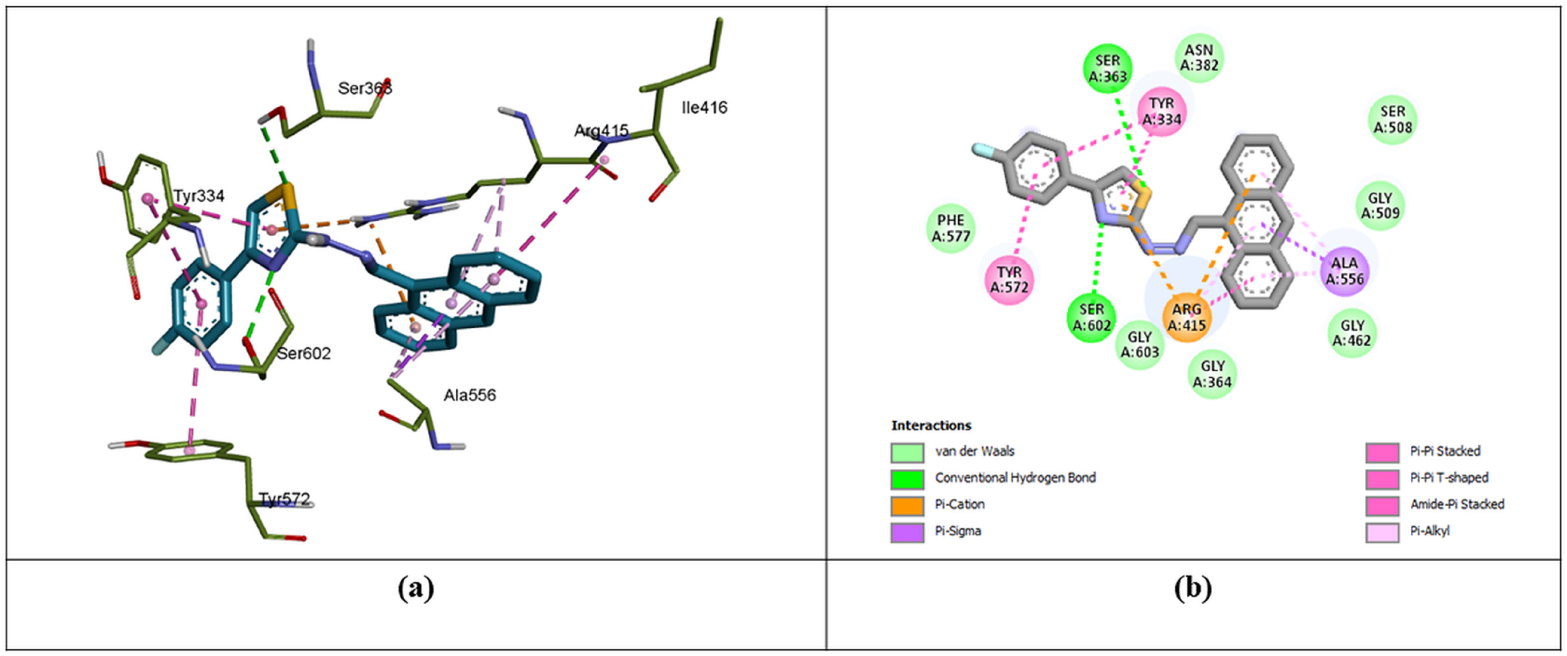
Predicted binding pose and two‐dimensional interaction map of **MM‐7** in the KEAP1 Kelch‐domain pocket.

**FIGURE 12:**
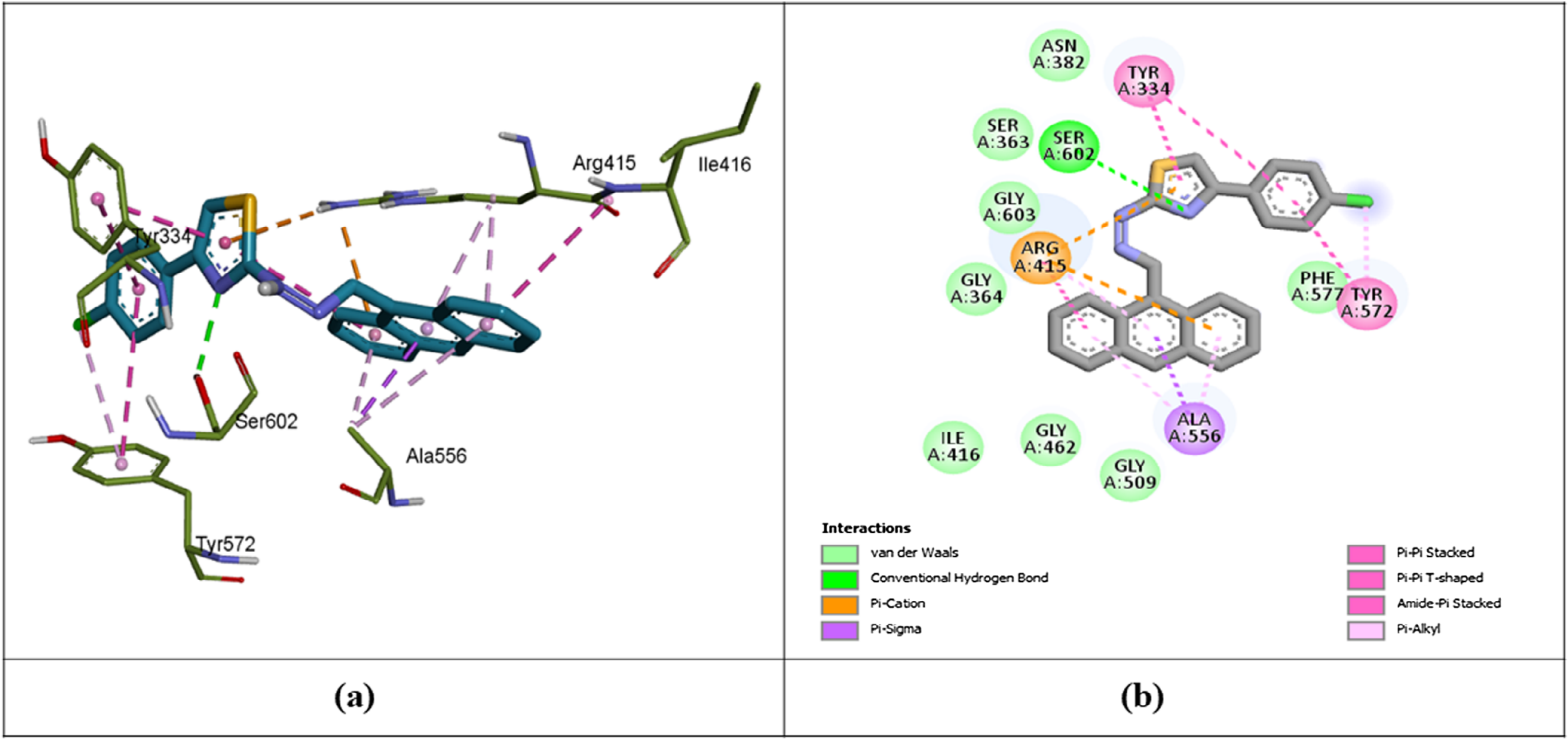
Predicted binding pose and two‐dimensional interaction map of **MM‐9** in the KEAP1 Kelch‐domain pocket.

**FIGURE 13:**
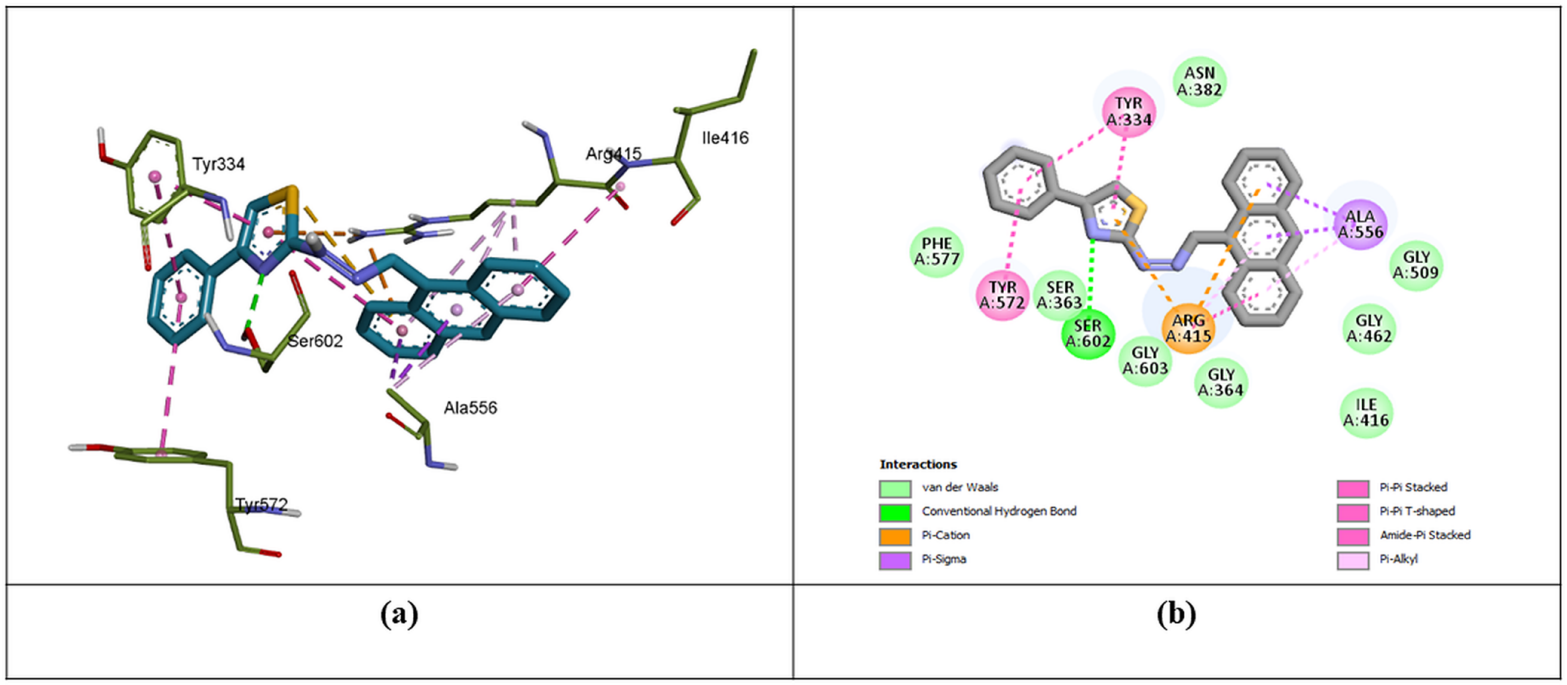
Compound **MM‐10** against *Keap‐1/Nrf2 complex*, amino acids colored by green, and the ligand colored by turquaz.

**FIGURE 14:**
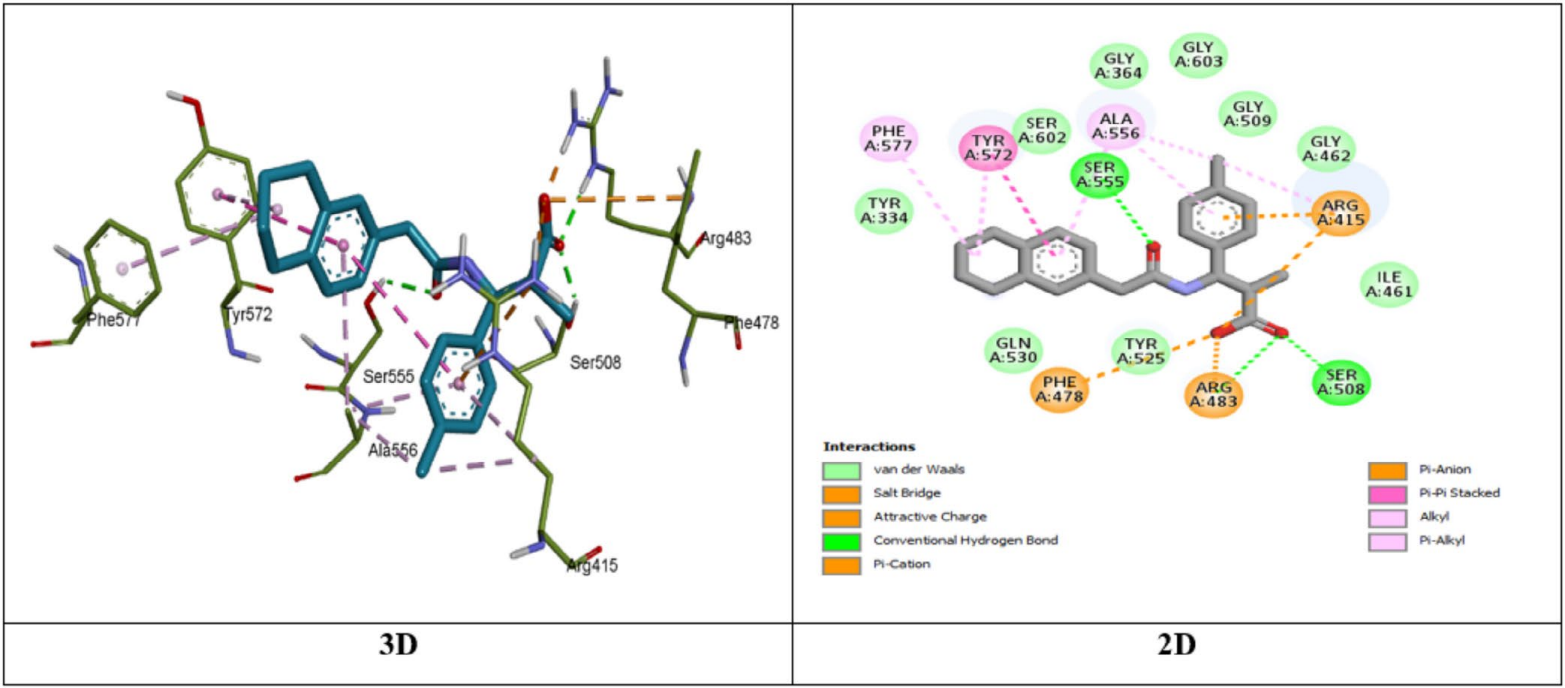
**Co‐crystalized ligand** complexed with *Keap‐1/Nrf2 complex*, amino acids colored green, and the co‐crystallized ligand colored by turquas.

Within **MM‐1‐MM‐5**, **MM‐3** has the smallest calculated HOMO‐LUMO gap (0.91 eV), largely because its two cyano and two carbonyl groups stabilize the LUMO. This narrow gap indicates enhanced electronic polarizability but does not imply superior radical‐scavenging ability; experimentally, **MM‐3** shows only intermediate DPPH activity. By contrast, **MM‐9** and **MM‐10** combine higher HOMO energies with lower electronegativity and electrophilicity and higher nucleophilicity values. These donor‐type descriptors are more consistent with their low experimental IC50 values. Accordingly, the calculated parameters are used to rationalize trends within the present series rather than to establish a universally predictive antioxidant model.

Between **MM‐4** and **MM‐5**, the calculated energy gaps and nucleophilicity values differ only modestly and do not reproduce the experimental activity ranking by themselves. These compounds therefore further illustrate why no single global descriptor should be interpreted as a direct predictor of DPPH potency.

Within **MM‐6‐MM‐10**, **MM‐9** and **MM‐10** display the most favorable donor‐type descriptor profiles, whereas **MM‐6‐MM‐8** occupy an intermediate range. The calculated trend is interpreted in conjunction with the experimental IC_50_ values and the systematic SAR analysis; individual orbital energies are not used as stand‐alone evidence of antioxidant efficacy.

#### 3.3.2 Molecular Electrostatic Potential (MEP) Surface Analysis

To learn how **MM‐1‐10** acts, referring to molecular electrostatics, MEPs were applied (Ayyaz et al. 2024). It is usually recognized that the ICT of each of the structures is a crucial property that may be determined with the support of MEPs. The MEP for each and every individual compound was evaluated, as shown in Figures 6 and 7. The electrical potential increased in the following order: red, orange, yellow, green, and blue. Nucleophilic‐attack‐prone regions, which are electron‐rich, and electrophilic‐attack‐prone regions, which are electron‐poor, are symbolized by the red (negative potential) and blue (positive potential) parts of the MEP surface, respectively. The compounds (**MM‐1‐5**) have two donor parts: anthracene and 4‐methylbenzene, 4‐methoxybenzene, anthracene, indole and 9‐ethylcarbazole, respectively. The carbonyl (CO) and cyano (CN) groups of the cyanoacetamide moiety were detected in all five compounds. The negative (red) low potentials were the most noticeable for these compounds.

The five compounds (**MM‐6‐10**) were positioned in the positive zone of the MEP map, symbolized by the color blue, above the donor moieties. All compounds (**MM‐6‐10**) composed of anthracene and hydrazo groups as donor parts with different acceptor parts. Furthermore, they possess a low potential for (**MM‐6‐10**), which has been centralized in the thiazole ring. But for compound **MM‐6**, has a thiazole ring conjugated with a thiophene ring bearing a Br atom. In addition, compounds (**MM‐7**) and **MM‐9** have a red region in the thiazole ring‐conjugated benzene ring bearing F and Cl atoms. The electron density transfer demonstrated, with visible positive and negative zones, that the molecules displayed an electron density distribution.

Beyond the qualitative description above, the MEP maps of **MM‐1** to **MM‐10** (Figures 6 and 7) can be interpreted mechanistically and mapped onto the two operative radical‐scavenging pathways. The electron‐rich (red, negative‐potential) regions correspond to the candidate single‐electron‐transfer (SET) donor sites for the DPPH^•^ radical. In **MM‐1** to **MM‐5**, these red lobes localize on the carbonyl oxygen and cyano‐nitrogen lone pair of the cyanoacrylamide moiety, together with the π‐cloud of the anthracene core. In **MM‐6** to **MM‐10**, the negative‐potential regions are distributed over the hydrazinyl N–N backbone and over the N and S heteroatoms of the thiazole ring, with additional small red lobes in the vicinity of the para‐halogen substituents (F in **MM‐7** and Cl in **MM‐9**) and the bromothiophene sulfur in **MM‐6**. The electron‐poor (blue, positive‐potential) regions identify the hydrogen atom transfer (HAT) donor sites. Both subseries display N‐H positive‐potential lobes: in **MM‐1** to **MM‐5**, the blue locus sits on the amide N‐H of the cyanoacrylamide linker, whereas in **MM‐6** to **MM‐10**, it sits on the hydrazinyl N‐H. The hydrazinyl N‐H is intrinsically more acidic than the amide N–H because the adjacent hydrazone nitrogen withdraws electron density from the N‐H bond (α‐N effect), and the resulting nitrogen‐centered radical is stabilized by spin delocalization across the hydrazinyl N–N system and into the thiazole ring. Therefore, the hydrazinyl‐thiazole series offers a better‐poised HAT donor than the cyanoacrylamide series, in accordance with the markedly lower IC_50_ values of **MM‐6** to **MM‐10**. Within the hydrazinyl‐thiazole subseries, the blue N‐H lobe is most clearly rendered in **MM‐6**, **MM‐8**, and **MM‐9** (Figure 7), where it coexists with a clear negative‐potential region on the hydrazinyl‐thiazole donor backbone. The MEP of **MM‐10** is comparatively more uniform; its top‐ranking experimental potency (IC_50_ = 0.0033 ± 0.0002 mg/mL) is therefore not driven by a visually striking MEP feature but by its descriptor profile, namely the highest E_HOMO_ and Nu and the lowest ω of the series, as captured quantitatively by the descriptor‐activity correlation analysis (Table 3, Figure 9), in which the nucleophilicity index Nu is identified as the strongest predictor of antioxidant potency. Within this framework, the para‐substituent effects observed for **MM‐9** (para‐Cl, lone‐pair delocalization of the post‐HAT radical by mesomeric resonance) and **MM‐10** (para‐Ph, extended π‐conjugation that raises the electron density on the hydrazinyl backbone and stabilizes the same radical) account for the trend within the most active subset of the series.

### 3.4 DPPH Radical Scavenging Activity

The DPPH radical‐scavenging activity **of MM‐1‐MM‐10** was measured over eight concentrations (0.003–0.417 mg/mL) in four independent experiments. The IC_50_ values ranged from 0.0033 ± 0.0002 to 0.0877 ± 0.0067 mg/mL (Table 2, Figure 8a,b). **MM‐10** and **MM‐9** were the most active derivatives, with IC_50_ values of 0.0033 ± 0.0002 and 0.0053 ± 0.0003 mg/mL, respectively, and both were significantly more active than ascorbic acid after Holm correction (adjusted *p* < 0.001). **MM‐7** was also significantly more active than ascorbic acid (0.0123 ± 0.0006 versus 0.0164 ± 0.0006 mg/mL; adjusted *p* < 0.001), whereas **MM‐6** was statistically comparable to the reference (0.0162 ± 0.0006 mg/mL; adjusted *p* = 0.654). **MM‐1‐MM‐5** and **MM‐8** had significantly higher IC_50_ values than ascorbic acid (adjusted *p* < 0.001). All compounds showed concentration‐dependent scavenging within the tested range.

**TABLE 2:** DPPH IC_50_ values of the synthesized derivatives (mean ± SD, *n* = 4) and statistical comparisons with ascorbic acid. Pairwise tests used two‐sided Welch's *t*‐tests with Holm correction.

Compound	IC_50_ (mg/mL), mean ± SD	Statistical comparison vs ascorbic acid
**MM‐1**	0.0413 ± 0.0019	Significantly higher IC50 (adjusted *p* < 0.001)
**MM‐2**	0.0770 ± 0.0038	Significantly higher IC50 (adjusted *p* < 0.001)
**MM‐3**	0.0505 ± 0.0016	Significantly higher IC50 (adjusted *p* < 0.001)
**MM‐4**	0.0707 ± 0.0033	Significantly higher IC50 (adjusted *p* < 0.001)
**MM‐5**	0.0877 ± 0.0067	Significantly higher IC50 (adjusted *p* < 0.001)
**MM‐6**	0.0162 ± 0.0006	Not significant (adjusted *p* = 0.654)
**MM‐7**	0.0123 ± 0.0006	Significantly lower IC50 (adjusted *p* < 0.001)
**MM‐8**	0.0258 ± 0.0012	Significantly higher IC50 (adjusted *p* < 0.001)
**MM‐9**	0.0053 ± 0.0003	Significantly lower IC50 (adjusted *p* < 0.001)
**MM‐10**	0.0033 ± 0.0002	Significantly lower IC50 (adjusted *p* < 0.001)
**Ascorbic acid**	0.0164 ± 0.0006	Reference

A clear family‐level SAR emerged from the DPPH data. The hydrazinyl‐thiazole derivatives **MM‐6‐MM‐10** were generally more active than the cyanoacrylamides **MM‐1‐MM‐5**, supporting the original design hypothesis (Negm et al. 2024; Salar et al. 2019). The hydrazinyl‐thiazole framework provides an accessible N‐H donor and permits stabilization of the resulting nitrogen‐centered species across the N‐N‐thiazole system, while the anthracene core contributes extended π‐delocalization. Within **MM‐6‐MM‐10**, the activity order was **MM‐10** (phenyl) > **MM‐9** (4‐chlorophenyl) > **MM‐7** (4‐fluorophenyl) > **MM‐6** (5‐bromothiophen‐2‐yl) > **MM‐8** (arylazo/methyl). The superior activity of **MM‐10** and **MM‐9** is consistent with their favorable donor‐type DFT profiles and with efficient delocalization across the aryl‐thiazole‐hydrazinyl framework. The weaker activity of **MM‐8** suggests that extended conjugation alone is insufficient when the substituent pattern reduces the availability of the hydrogen‐donating site or shifts the electronic profile toward poorer donation.

Within **MM‐1‐MM‐5**, **MM‐1** was the most active, followed by **MM‐3**, **MM‐4**, **MM‐2**, **and MM‐5**. The bis‐anthracene system of **MM‐3** increases conjugation and gives the smallest calculated gap, but its strong electrophilic character limits its DPPH performance. Similarly, Jin et al. (2013) and Assefa et al. (2023) reported that electron‐rich aromatic systems and hydrogen‐donating groups augmented the radical‐scavenging activities of DPPH. The methoxyphenyl, indolyl, and carbazolyl groups do not produce a simple monotonic donor‐substituent trend, indicating that steric accessibility, linker polarization, and the ability to stabilize the post‐transfer species act together. Thus, the experimental SAR is best explained by the combined effects of an accessible H‐donor, donor‐type electronic descriptors, and radical delocalization rather than by any single substituent constant or by the HOMO‐LUMO gap alone.

#### 3.4.1 Exploratory Correlation Between DFT Descriptors and DPPH Activity

To explore the relationship between the computed electronic descriptors and DPPH potency, pIC50 values were correlated with E_HOMO_, E_LUMO_, Eg, *χ*, *μ*, *η*, *ω*, *σ*, and Nu calculated at the B3LYP/6‐311G(d, p) level. Pearson coefficients and two‐tailed *p* values were calculated for the 10 compounds, with Spearman rank coefficients used as a complementary nonparametric measure. Because the analysis contains only 10 structurally related compounds and multiple correlated descriptors, it is explicitly considered exploratory and hypothesis‐generating; no external validation or claim of general predictive performance is made.

Pearson correlation coefficients (*r*) and associated two‐tailed *p*‐values were computed across the 10 anthracene derivatives (*n* = 10). The results are summarized in Table 3 and illustrated in Figure 9.

**TABLE 3:** Correlation between DPPH antioxidant potency (pIC_50_) and DFT‐derived quantum chemical descriptors of compounds **MM‐1**‐**MM‐10**.

Descriptor	Pearson *r* vs. pIC_50_	*p*‐value	Spearman *ρ* vs. IC_50_	Mechanistic interpretation
E_HOMO	+0.702	0.024*	−0.224	Higher (less negative) HOMO → easier electron donation → stronger antioxidants.
E_LUMO	+0.970	< 0.001***	−0.842	Strong covariation with HOMO across the series tracks the donor‐acceptor electronic asymmetry.
E_g	+0.618	0.057 n.s.	−0.648	The gap alone is not significant and does not discriminate between the donor and acceptor characteristics.
*χ*	−0.908	< 0.001***	+0.818	Lower electronegativity → better electron donor → stronger antioxidants.
*μ*	+0.908	< 0.001***	−0.818	A less negative chemical potential results in easier oxidation and stronger antioxidants.
*η*	+0.615	0.059 n.s.	−0.640	Borderline trend only; hardness alone does not capture donor selectivity.
*ω*	−0.837	0.003**	+0.770	Lower electrophilicity → the molecule preferentially donates rather than accepts electrons → better antioxidant activity.
*σ*	−0.516	0.127 n.s.	+0.648	Softness loosely tracks reactivity, but lacks donor/acceptor selectivity.
Nu	+0.887	< 0.001***	−0.793	The strongest mechanistically meaningful predictor is higher nucleophilicity → greater electron‐donating capacity → stronger DPPH scavenging.

*Note:* Pearson r vs. pIC_50_ with two‐tailed *p*‐values; Spearman *ρ* vs. IC_50_ provided as a rank‐based control.

Abbreviation: n.s. = not significant.

*** *p* < 0.001.

** *p* < 0.01.

* *p* < 0.05.

Within this limited series, donor‐related descriptors showed stronger associations with DPPH potency than the HOMO‐LUMO gap considered in isolation. Nu showed a positive correlation with pIC_50_ (*r* = +0.887, *p* < 0.001), whereas lower electronegativity and electrophilicity were associated with greater potency. Eg did not reach conventional statistical significance (*r* = +0.618, *p* = 0.057). These results support the interpretation that electron‐ or hydrogen‐donating capacity is more relevant to the observed DPPH trend than global softness alone. However, the small sample size, structural clustering of the two subseries, and interdependence of the descriptors preclude treating these relationships as a validated predictive model.

**MM‐3** illustrates the limitation of relying on Eg alone. Although it has the smallest calculated gap (0.91 eV), it also has the highest electrophilicity and one of the lowest nucleophilicity values, consistent with only intermediate DPPH activity. Conversely, **MM‐9** and **MM‐10** combine the highest HOMO energies, the lowest electronegativity and electrophilicity values, and the highest Nu values in the series, in agreement with their strong radical‐scavenging performance. This internal consistency supports the proposed donor‐capacity hypothesis but remains specific to the present, small compound set.

### 3.5 Molecular Docking Simulation of Target Compounds Against the KEAP1/Nrf2 Complex

KEAP1 was selected as a pathway‐relevant docking target because the KEAP1‐Nrf2 interaction regulates endogenous cellular antioxidant responses (Ngo and Duennwald 2022). This rationale is complementary to the DPPH assay but does not create a direct experimental link between the two measurements. DPPH quantifies chemical radical scavenging in solution, whereas docking estimates the geometric and energetic compatibility of a ligand with the KEAP1 Kelch pocket. The co‐crystallized inhibitor SIU was redocked with an RMSD of 0.23 A and was retained as the reference ligand. Thus, the docking analysis is used to prioritize compounds for future KEAP1 binding and Nrf2 activation studies, not to claim biological antioxidant efficacy. The same co‐crystal ligand was retained throughout the analysis as the known‐inhibitor reference, against which the affinities and CNN scores of the **MM‐1 to MM‐10** series were compared (Table 4, Figure 14). Among the designed compounds, several **MM‐1 to MM‐10** ligands achieved binding affinities and CNN scores comparable to or better than those of the co‐crystal reference inhibitor (Table 4). In terms of classical docking energy, **MM‐3** (−10.11 kcal/mol) and **MM‐7** (−9.94 kcal/mol) outperformed the co‐crystal ligand (−9.73 kcal/mol), whereas **MM‐8** (−9.39 kcal/mol), **MM‐9** (−9.48), and **MM‐10** (−9.52 kcal/mol) also resided in a similar high‐affinity range. The **MM‐3**–KEAP1 complex was stabilized by three hydrogen bonds (with Asn414, Arg415, and Ser602), three pi‐cation interactions, and additional π‐sigma, π‐π, π‐amide, and pi‐alkyl contacts (Figure 10), whereas the **MM‐7**–KEAP1 complex involved two hydrogen bonds (with Ser363 and Ser602) together with π‐cation, π‐sigma, π‐π, π‐amide, and π‐alkyl contacts (Figure 11). Similar interaction profiles were observed for **MM‐**9 and **MM‐10**, which both engage the KEAP1 Kelch domain through a combination of hydrogen bonding and π‐cation, π‐π, and π‐alkyl contacts (Figures 12 and 13, respectively). CNN‐based evaluation provided additional discrimination: **MM‐5** exhibited the highest CNN affinity (7.11), followed by **MM‐3** (6.79), **MM‐7** (6.52), **MM‐8** (6.39), and **MM‐9** (6.37), all exceeding the co‐crystal value of 5.79 and indicating favorable interaction patterns within the KEAP1 pocket. Inspection of the CNN pose scores, which reflect the confidence in the predicted geometry, highlighted **MM‐7** (0.89), **MM‐10** (0.88), and **MM‐9** (0.87) as particularly reliable poses, closely approaching the co‐crystal ligand's confidence level (0.92). Overall, **MM‐3**, **MM‐7**, **MM‐9**, and **MM‐10** emerged as the most promising KEAP1 binders, combining favorable docking affinities with strong CNN scores and high‐confidence binding poses comparable to those of the validated co‐crystal inhibitor (Figures 10, 11, 12, 13, 14). **MM‐3 and MM‐7** gave the most favorable classical docking scores (−10.11 and −9.94 kcal/mol, respectively), compared with −9.73 kcal/mol for SIU. MM‐9 and MM‐10 also occupied the reference pocket with predicted affinities of −9.48 and −9.52 kcal/mol and high CNN pose scores (0.87 and 0.88). The predicted poses included hydrogen‐bonding and aromatic/hydrophobic contacts with residues lining the Kelch‐domain pocket. These results indicate that MM‐3, MM‐7, MM‐9, and MM‐10 are reasonable candidates for experimental target‐engagement studies.

**TABLE 4:** Molecular docking results of the compound series **MM‐1‐10** with **the KEAP1** protein.

Ligand	Affinity (kcal/mol)	CNN affinity	CNN pose score
MM‐1	−8.86	5.89	0.66
MM‐2	−8.56	5.97	0.75
MM‐3	−10.11	6.79	0.52
MM‐4	−9.29	6.25	0.81
MM‐5	−8.79	7.11	0.83
MM‐6	−8.60	5.98	0.69
MM‐7	−9.94	6.52	0.89
MM‐8	−9.39	6.39	0.78
MM‐9	−9.48	6.37	0.87
MM‐10	−9.52	6.12	0.88
Co‐crystal inhibitor	−9.73	5.79	0.92

## 4 Conclusion

Ten anthracene derivatives belonging to cyanoacrylamide and hydrazinyl‐thiazole subseries were synthesized and characterized. DPPH testing identified **MM‐10**, **MM‐9**, **and MM‐7** as the strongest radical scavengers, while MM‐6 showed activity comparable to ascorbic acid. The family‐level comparison supports the hydrazinyl‐thiazole linkage as a more favorable design element than the cyanoacrylamide linker for this assay. DFT calculations and an exploratory descriptor‐activity analysis provided a consistent qualitative rationale: donor‐related descriptors were more informative for the observed trend than the HOMO‐LUMO gap alone. Docking to the KEAP1 Kelch domain generated plausible poses for **MM‐3**, **MM‐7**, **MM‐9**, **and MM‐10** relative to the co‐crystallized inhibitor, but these predictions are supportive only and do not establish pathway modulation. Accordingly, **MM‐7**, **MM‐9**, **and MM‐10** should be regarded as lead scaffolds for further investigation rather than validated biological antioxidants. Cellular radical‐stress models, direct KEAP1/Nrf2 target‐engagement assays, cytotoxicity testing, pharmacokinetic assessment, and in vivo studies are required before biological or therapeutic conclusions can be drawn.

## Author Contributions

**Mohamed R. Elmorsy:** supervision, methodology, investigation, formal analysis. **Menna Moustafa:** investigation, methodology, software, formal analysis. **Hassan A. Etman:** writing – original draft, investigation, validation, formal analysis, supervision. **Nashwa M. El‐Metwaly:** funding acquisition, methodology, software. **Safa A. Badawy:** conceptualization, writing – original draft, formal analysis, methodology, supervision.

## Funding

This research work was funded by Umm Al‐Qura University, Saudi Arbia Under grant number: (26UQU4350527GSSR13).

## Conflicts of Interest

The authors declare no conflicts of interest.

## Supporting information


**Figure S1:** IR spectrum of compound MM‐1.
**Figure S2:** 1H NMR spectrum of compound MM‐1.
**Figure S3:** Mass spectrum of compound MM‐1.
**Figure S4:** IR spectrum of compound MM‐2.
**Figure S5:** 1H NMR spectrum of compound MM‐2.
**Figure S6:** 13C NMR spectrum of compound MM‐2.
**Figure S7:** Mass spectrum of compound MM‐2.
**Figure S8:** IR spectrum of compound MM‐3.
**Figure S9:** 1H NMR spectrum of compound MM‐3.
**Figure S10:** Expanded ^1^H NMR spectrum of compound MM‐3.
**Figure S11:** 13C NMR spectrum of compound MM‐3.
**Figure S12:** Mass spectrum of compound MM‐3.
**Figure S13:** IR spectrum of compound MM‐4.
**Figure S14:** 1H NMR spectrum of compound MM‐4.
**Figure S15:** 13C NMR spectrum of compound MM‐4.
**Figure S16:** Mass spectrum of compound MM‐4.
**Figure S17:** IR spectrum of compound MM‐5.
**Figure S18:** 1H NMR spectrum of compound MM‐5.
**Figure S19:** Mass spectrum of compound MM‐5.
**Figure S20:** IR spectrum of compound MM‐6.
**Figure S21:** 1H NMR spectrum of compound MM‐6.
**Figure S22:** ^13^C NMR spectrum of compound MM‐6.
**Figure S23:** Mass spectrum of compound MM‐6.
**Figure S24:** IR spectrum of compound MM‐7.
**Figure S25:** 1H NMR spectrum of compound MM‐7.
**Figure S26:** 13C NMR spectrum of compound MM‐7.
**Figure S27:** Mass spectrum of compound MM‐7.
**Figure S28:** IR spectrum of compound MM‐8.
**Figure S29:** 1H NMR spectrum of compound MM‐8.
**Figure S30:** Expanded ^1^H NMR spectrum of compound MM‐8.
**Figure S31:** 13C NMR spectrum of compound MM‐8.
**Figure S32:** Mass spectrum of compound MM‐8.
**Figure S33:** IR spectrum of compound MM‐9.
**Figure S34:** 1H NMR spectrum of compound MM‐9.
**Figure S35:** 13C NMR spectrum of compound MM‐9.
**Figure S36:** Mass spectrum of compound MM‐9.
**Figure S37:** IR spectrum of compound MM‐10.
**Figure S38:** 1H NMR spectrum of compound MM‐10.
**Figure S39:** 13C NMR spectrum of compound MM‐10.
**Figure S40:** Mass spectrum of compound MM‐10.

## Data Availability

The data supporting the findings of this study are available in the Supporting Information of this article.
