# Supplementary material for: Design, Synthesis, and Biological Evaluation of Anthracene Derivatives as Potent Antioxidants: Experimental, DFT, and Docking Studies
Source: Chem Biol Drug Des. 2026 Sep 6;108(3):e70392. doi: 10.1111/cbdd.70392 (PMC13547762; doi:10.1111/cbdd.70392)
Supplement: Supplementary file 1 [file CBDD-108-e70392-s001.docx]

1. **Materials**

All starting materials, including anthracene-9-carbaldehyde, 1-cyanoacetyl-3,5-dimethylpyrazole, *p*-toluidine, *p*-anisidine, 2-cyano-*N′*-(2-cyanoacetyl)acetohydrazide, 1*H*-indol-3-amine, 9-ethyl-9*H*-carbazol-3-amine, hydrazinecarbothioamide, 2-bromo-1-(5-bromothiophen-2-yl)ethan-1-one, 2-chloro-1-(4-fluorophenyl)ethan-1-one, *N*-(4-chlorophenyl)-2-oxopropanehydrazonoyl chloride, 2-bromo-1-(4-chlorophenyl)ethan-1-one, and 2-bromo-1-phenylethan-1-one, were purchased from Alfa Aesar and Sigma-Aldrich and used without further purification unless otherwise stated. All solvents were dried prior to use according to standard procedures. Melting points were determined in degrees Celsius using a Gallenkamp electric melting point apparatus and are uncorrected. Infrared spectra were recorded as KBr pellets using a Thermo-Scientific Nicolet iS10 FTIR spectrometer. Nuclear magnetic resonance spectra were recorded in DMSO-*d*₆ using a Bruker NMR spectrometer operating at 400 MHz for ¹H NMR and 100 MHz for ¹³C NMR. Chemical shifts are reported in ppm relative to tetramethylsilane (TMS) as an internal standard. Mass spectra were recorded to confirm the molecular ion peaks and fragmentation patterns of the synthesized compounds.

Figure S1: IR spectrum of compound MM-1.

Figure S2: 1H NMR spectrum of compound MM-1.

Figure S3: Mass spectrum of compound MM-1.

Figure S4: IR spectrum of compound MM-2.

Figure S5: 1H NMR spectrum of compound MM-2.

Figure S6: 13C NMR spectrum of compound MM-2.

Figure S7: Mass spectrum of compound MM-2.

Figure S8: IR spectrum of compound MM-3.

Figure S9: 1H NMR spectrum of compound MM-3.

Figure S10: Expanded ¹H NMR spectrum of compound MM-3.

Figure S11: 13C NMR spectrum of compound MM-3.

Figure S12: Mass spectrum of compound MM-3.

Figure S13: IR spectrum of compound MM-4.

Figure S14: 1H NMR spectrum of compound MM-4.

Figure S15: 13C NMR spectrum of compound MM-4.

Figure S16: Mass spectrum of compound MM-4.

Figure S17: IR spectrum of compound MM-5.

Figure S18: 1H NMR spectrum of compound MM-5.

Figure S19: Mass spectrum of compound MM-5.

Figure S20: IR spectrum of compound MM-6.

Figure S21: 1H NMR spectrum of compound MM-6.

Figure S22: ¹³C NMR spectrum of compound MM-6.

Figure S23: Mass spectrum of compound MM-6.

Figure S24: IR spectrum of compound MM-7.

Figure S25: 1H NMR spectrum of compound MM-7.

Figure S26: 13C NMR spectrum of compound MM-7.

Figure S27: Mass spectrum of compound MM-7.

Figure S28: IR spectrum of compound MM-8.

Figure S29: 1H NMR spectrum of compound MM-8

Figure S30: Expanded ¹H NMR spectrum of compound MM-8.

Figure S31: 13C NMR spectrum of compound MM-8.

Figure S32: Mass spectrum of compound MM-8.

**Figure S33: IR spectrum of compound MM-9.**

Figure S34: 1H NMR spectrum of compound MM-9.

Figure S35: 13C NMR spectrum of compound MM-9.

Figure S36: Mass spectrum of compound MM-9.

Figure S37: IR spectrum of compound MM-10.

Figure S38: 1H NMR spectrum of compound MM-10.

Figure S39: 13C NMR spectrum of compound MM-10.

Figure S40: Mass spectrum of compound MM-10.

Computational details. The geometries of MM-1-MM-10 were optimized in the gas phase with Gaussian 09W at the B3LYP/6-311G(d,p) level. Harmonic frequency calculations were performed at the same level, and no imaginary frequencies were obtained, confirming that all structures correspond to local minima. The Cartesian coordinates listed below are those of the optimized structures used for the frontier-orbital, molecular-electrostatic-potential, and global-reactivity-descriptor analyses.

**Cartesian coordinates of the optimized compounds MM-1-MM-10**

Cartesian coordinates for compound MM-1.

C	4.26799629	-3.47329029	-0.16267432

C	2.78624269	-3.50546822	0.25639011

C	2.81578702	-0.99706316	0.21644447

C	4.12110878	-0.96871740	-0.15271888

C	4.89283752	-2.28596026	-0.35417125

C	2.04430264	0.32152605	0.41779920

C	2.67600837	1.50373355	0.22441681

C	4.14371954	1.53560480	-0.19066611

C	4.83010578	0.38202083	-0.37005877

C	1.94375140	2.83825062	0.41448980

C	2.60432551	4.01194462	0.21306979

C	4.09796876	4.04437846	-0.20934340

C	4.81278784	2.90055081	-0.39689852

C	0.56262341	0.29513056	0.83675700

C	-0.28586483	-0.59702839	0.27046760

N	-2.61666505	-1.06679495	-0.79864883

C	-3.93665184	-0.50502084	-0.47779733

C	-5.19173791	-1.39387653	-0.55713035

C	-6.40863617	-0.87597315	-0.26132177

C	-6.53638966	0.60140943	0.15415399

C	-5.43191464	1.38360457	0.22396017

C	-4.04907570	0.79507760	-0.11218375

C	-7.91922877	1.18993238	0.49030533

C	0.03439496	-2.10255245	0.31977521

N	0.27284254	-3.22348346	0.35649186

H	4.80423154	-4.38858035	-0.30265935

H	2.29448700	-4.44305318	0.41137630

H	5.92223410	-2.26386695	-0.64529732

H	5.85939542	0.40839366	-0.66120637

H	0.91406577	2.84688090	0.70530436

H	2.08075400	4.93508869	0.34937041

H	4.57781636	4.98931116	-0.35681636

H	5.84102318	2.95386772	-0.68808456

H	0.20307332	0.98118972	1.57496226

H	-2.48467057	-1.91852105	-0.29155942

H	-5.10297455	-2.42036997	-0.84580323

H	-7.28067587	-1.49355622	-0.31643621

H	-5.52067824	2.41009893	0.51263077

H	-3.17703527	1.41265969	-0.05706408

H	-7.79551788	2.11284069	1.01740686

H	-8.46150036	1.36621655	-0.41510369

H	-8.46147158	0.49964904	1.10217261

C	-1.57031270	-0.10531037	-0.42235960

O	-1.76921251	1.10879722	-0.68684442

C	2.20283160	-2.32883623	0.68755576

H	1.30602164	-2.81107984	1.14709405

Cartesian coordinates for compound MM-2.

C	4.66403499	-3.44567311	0.00747861

C	3.16444104	-3.48590662	0.35653982

C	3.17310811	-0.97824755	0.27820553

C	4.49414583	-0.94280532	-0.02928986

C	5.28646101	-2.25537037	-0.17311979

C	2.38102941	0.33566453	0.42192810

C	3.01044772	1.52088379	0.23985220

C	4.49582972	1.56073444	-0.10589092

C	5.20037311	0.41130461	-0.23431879

C	2.25794100	2.85093155	0.37390709

C	2.91665210	4.02779775	0.18530240

C	4.42827822	4.06835203	-0.16654585

C	5.16151968	2.92882969	-0.30193790

C	0.88146239	0.30121156	0.77079450

C	0.06888190	-0.60785758	0.17929631

N	-2.20428677	-1.11689939	-0.99113175

C	-3.54303192	-0.56320978	-0.74195212

C	-4.78484107	-1.46544745	-0.86635785

C	-6.01903347	-0.95499695	-0.63662377

C	-6.17971612	0.52729839	-0.25115997

C	-5.08692447	1.32126956	-0.14168986

C	-3.68443324	0.74121180	-0.40274913

C	0.40007815	-2.10922892	0.26757871

N	0.64666817	-3.22706797	0.33331385

H	5.21457504	-4.35771288	-0.09246742

H	2.67441200	-4.42576459	0.50299340

H	6.32825373	-2.22768061	-0.41560943

H	6.24202123	0.44327179	-0.47690294

H	1.21559586	2.85395262	0.61558405

H	2.37886443	4.94775754	0.28204207

H	4.90599042	5.01555572	-0.30617471

H	6.20187235	2.98772552	-0.54503812

H	0.48113295	0.99502379	1.48020379

H	-2.08875442	-1.95936279	-0.46491182

H	-4.67319844	-2.49535428	-1.13417829

H	-6.88184855	-1.58187778	-0.72305492

H	-5.19856725	2.35117727	0.12612827

H	-2.82161762	1.36809174	-0.31631270

C	-1.18569781	-0.13950852	-0.58115206

O	-1.38283790	1.06836404	-0.87398392

C	2.55061379	-2.30853720	0.74092610

H	1.63744385	-2.79240117	1.16518896

C	-8.67667922	0.58556674	1.23810206

H	-9.21538866	1.48802092	1.61644463

H	-9.32320735	0.08767622	0.47509212

H	-8.53740083	-0.12111428	2.09207978

O	-7.35297386	0.96728401	0.64409359

Cartesian coordinates for compound MM-3.

C	1.19605940	-0.50279082	0.16502203

N	0.37319315	-1.53158555	0.02848049

O	0.68915536	0.63065582	0.05359295

N	-0.97812210	-1.18027935	-0.10353377

C	-1.92450397	-2.07603087	-0.30752248

C	-3.21528210	-1.67340782	-0.38494828

O	-1.63409394	-3.29502019	-0.43242406

C	-3.38991313	-0.34826570	-0.26363587

C	3.86474333	-0.84134480	0.20177499

C	4.97522239	-0.07753713	0.06685776

C	-4.59286738	0.27053722	-0.09574707

C	5.11670652	1.30046854	-0.15703080

C	6.36626754	1.91295740	-0.28425402

C	7.53062272	1.13665444	-0.19886042

C	7.41276326	-0.25207786	0.02340585

C	6.14305631	-0.83247281	0.15753777

C	8.55126962	-1.06208302	0.10192150

C	8.41630757	-2.44163653	0.31916090

C	7.14538362	-3.01068113	0.45937398

C	6.01501024	-2.20135848	0.37946060

C	3.95365567	3.57760308	-0.48106715

C	5.20302801	4.20733301	-0.61340877

C	6.40590001	3.36489596	-0.51715673

C	-5.85991459	-0.36292649	-0.13354236

C	-7.01825096	0.41729234	0.06874571

C	-6.90825058	1.79801960	0.30934810

C	-5.64840082	2.40632763	0.33759268

C	-4.50869456	1.63338311	0.12838639

C	-5.99003826	-1.74304009	-0.36834541

C	-7.26858364	-2.32933804	-0.40993147

C	-8.41398935	-1.54630502	-0.21209731

C	-8.28810794	-0.17434534	0.02995696

C	-5.51494529	3.78200255	0.57378145

C	-4.23685967	4.36708281	0.59623086

C	-3.10019407	3.57925320	0.38145532

C	-3.23866138	2.21360406	0.14742079

C	-4.23429420	-2.54714910	-0.20423284

N	-4.97956983	-3.42128512	-0.08646421

H	0.68038511	-2.48303926	0.03071419

H	-1.23843369	-0.21706793	-0.03867673

H	-2.51392791	0.24026506	-0.09441631

H	4.07057488	-1.82375626	0.54965723

H	8.49255301	1.59442468	-0.30709391

H	9.52323136	-0.62469449	-0.00304175

H	9.28147024	-3.05936297	0.37638433

H	7.03770034	-4.06197158	0.62808461

H	5.04117947	-2.62424058	0.48761439

H	3.06874783	3.99952111	-0.92642358

H	5.27981747	5.26208320	-0.77909032

H	7.36257996	3.83834909	-0.62118745

H	-7.78963496	2.38405708	0.46648409

H	-5.12024594	-2.34962745	-0.51963999

H	-7.37113183	-3.37798608	-0.59510776

H	-9.38594681	-1.99493646	-0.24408373

H	-9.16202516	0.42366619	0.18663011

H	-6.38197347	4.38497400	0.73950161

H	-4.13028826	5.41541002	0.77962793

H	-2.12695838	4.01996773	0.39400062

H	-2.37249810	1.60430924	-0.02028541

C	2.55292381	-0.53079420	0.07053787

C	2.40014935	-1.69092403	0.34190513

N	2.25261490	-2.81126252	0.60396476

C	4.23851359	2.28801054	-0.29261779

H	3.82253521	1.30996098	-0.16900130

Cartesian coordinates for compound MM-4.

C	3.27445867	4.02603338	-0.38556852

C	1.95925215	3.58889362	0.28502738

C	1.74872912	2.28308136	0.57839778

C	2.82754963	1.23455740	0.24797680

C	3.98600390	1.61943153	-0.34389429

C	4.22255480	3.10485610	-0.68065170

C	2.59384600	-0.25128577	0.58975914

C	3.54624203	-1.16589303	0.29008886

C	4.84803247	-0.73398424	-0.37775695

C	5.06408872	0.56929724	-0.67779505

C	3.34925964	-2.65417605	0.60862642

C	4.32289253	-3.55254539	0.29277945

C	5.64768913	-3.11339420	-0.38673351

C	5.89467407	-1.81061299	-0.69683779

C	1.18325090	-0.75237674	0.95154893

C	0.09550342	-0.03001611	0.59167266

C	-0.98709737	-0.66338229	-0.30238945

N	-2.15960993	0.12501535	-0.70498864

O	-0.86781608	-1.85580860	-0.68753973

C	-3.38594689	-0.61572255	-0.37641518

C	-3.49040146	-1.94429839	-0.12731369

N	-4.85016025	-2.26210164	0.38746590

C	-5.61048284	-1.03197483	0.05509086

C	-4.79097329	-0.04540396	-0.27734461

C	-7.12746959	-0.82640693	0.06438663

C	-7.60727722	0.42611664	-0.19078556

C	-6.62740449	1.60255825	-0.48237751

C	-5.28325830	1.38108071	-0.52706502

C	-0.05818226	1.42329515	1.07672437

N	-0.17608876	2.50608846	1.43609150

H	3.43941539	5.05754728	-0.61840504

H	1.21165694	4.31689214	0.51923087

H	0.83327555	1.98032270	1.03981649

H	5.13556229	3.40986974	-1.14749839

H	5.97865386	0.86853930	-1.14447109

H	2.44689194	-2.98643123	1.07852872

H	4.17099624	-4.58746756	0.51849604

H	6.38540947	-3.85313336	-0.61740321

H	6.81861325	-1.53843401	-1.16322473

H	1.06219306	-1.67131707	1.48629698

H	-2.15849100	0.99788243	-0.21766553

H	-2.71130691	-2.66103411	-0.28304283

H	-4.88023316	-3.20139932	0.72982799

H	-7.79128076	-1.63867904	0.27112338

H	-8.66262739	0.60179161	-0.18753557

H	-7.01115538	2.58653457	-0.65209775

H	-4.59723348	2.17560190	-0.73654245

Cartesian coordinates for compound MM-5.

C	-7.01649219	-1.97115017	1.27681175

C	-5.98009561	-2.67833422	0.38732151

C	-4.97970227	-1.96305075	-0.18034315

C	-4.87082788	-0.43257238	0.06308875

C	-5.77378899	0.20346586	0.84632089

C	-6.92603876	-0.62720047	1.49569418

C	-3.72285877	0.36434775	-0.59584486

C	-3.59177102	1.68724557	-0.39692724

C	-4.58956397	2.43105438	0.48125424

C	-5.62624560	1.74325329	1.06927713

C	-2.43362256	2.46407477	-1.04653039

C	-2.30271225	3.79924987	-0.83495084

C	-3.30759367	4.57588206	0.05149146

C	-4.36711938	3.95267791	0.66260943

C	-2.71649886	-0.38422700	-1.46555273

C	-2.11314284	-1.43413224	-0.90057077

C	-0.61265598	-1.53607970	-0.78570467

N	0.16171803	-0.38284166	-1.18366024

O	-0.04483735	-2.57862590	-0.36986575

C	1.55459590	-0.63390230	-0.91428282

C	2.47617788	0.57902398	-0.68865079

C	3.69612150	0.31905518	-0.23574456

C	4.17266271	-1.08105203	0.07041941

C	3.44801435	-2.15940989	-0.28745289

C	2.02816522	-1.90596349	-0.84086217

C	4.85380607	1.24148796	0.05849804

C	5.96113609	0.34157006	0.54831325

N	5.47066390	-1.05043991	0.78222036

C	4.99236472	2.57111519	-0.06559409

C	6.38066757	3.17364038	0.24720526

C	7.42307761	2.34382853	0.57968178

C	7.20263699	0.81479490	0.73746889

C	6.28252403	-2.06399156	0.13717749

C	7.24873658	-2.64927816	1.17485149

C	-2.97162053	-2.56845885	-0.31705310

N	-3.60754284	-3.40873654	0.11520146

H	-7.80724154	-2.54378619	1.72294725

H	-6.05335231	-3.73530263	0.22090393

H	-4.26091318	-2.45923569	-0.80089590

H	-7.65035674	-0.14479178	2.11633855

H	-6.33367145	2.26500767	1.68700549

H	-1.72808870	1.95066827	-1.66701106

H	-1.48603652	4.32495984	-1.28958139

H	-3.16348041	5.63172671	0.18916898

H	-5.03842937	4.52433584	1.26545498

H	-2.49067448	-0.07802727	-2.46500629

H	-0.15372947	0.42800808	-0.67174426

H	2.15527897	1.58331752	-0.87681260

H	3.82915866	-3.15049845	-0.19503521

H	1.41493001	-2.72360078	-1.14450802

H	4.17510335	3.19159504	-0.37152922

H	6.53084383	4.23293613	0.19693297

H	8.39919943	2.75237269	0.74058632

H	8.00644104	0.16318355	0.99990649

H	5.65329148	-2.83233009	-0.26753898

H	6.84628546	-1.61585487	-0.65612631

H	7.88469207	-3.36024905	0.70887246

H	7.84271906	-1.86628519	1.58208791

H	6.70283944	-3.11680189	1.94965963

Cartesian coordinates for compound MM-6.

C	-5.07837040	-2.44397341	-0.13959219

C	-3.64374671	-2.28746269	0.40056391

C	-3.09555255	-1.05202034	0.51938229

C	-3.90842187	0.19269212	0.11398870

C	-5.16853485	0.04504217	-0.35969805

C	-5.78492621	-1.34409373	-0.49329799

C	-6.01071470	1.25843790	-0.77402612

C	-7.27516639	1.07994862	-1.24708943

C	-7.90244766	-0.33373007	-1.38305067

C	-7.21561665	-1.45700109	-1.03518428

C	-2.83212135	-3.53189286	0.80545379

C	-1.57040556	-3.39277440	1.28039578

C	-0.94811536	-1.99034539	1.41527386

C	-1.66193954	-0.89470058	1.05908808

C	-3.29109208	1.59727997	0.24679368

N	-2.08714469	1.73447321	0.69966215

C	-0.06924968	2.92518102	0.64288297

N	0.67796667	3.91923671	0.23230656

C	2.06908933	3.47837393	-0.08521397

C	2.28207092	2.15836741	0.14868037

S	0.87909414	1.46393168	0.94849933

C	3.55753735	1.38489850	-0.23411384

C	4.76068696	1.95944713	-0.50104716

C	5.88850276	0.90566535	-0.66400074

C	5.42972114	-0.36439921	-0.50466929

S	3.66284317	-0.37701884	-0.38153623

Br	6.53735159	-1.91803532	-0.41824141

N	-1.52594429	3.01137460	0.82039821

H	-5.51406289	-3.41661074	-0.23471670

H	-5.60466485	2.24493941	-0.69133206

H	-7.85089792	1.93557702	-1.53227933

H	-8.89958088	-0.42780134	-1.75958211

H	-7.67382707	-2.41824795	-1.13981551

H	-3.26421207	-4.50628220	0.71184238

H	-1.00644576	-4.25746875	1.56173724

H	0.04803545	-1.88103292	1.79028635

H	-1.22940892	0.07947567	1.15288494

H	-3.85801354	2.45971726	-0.03552166

H	2.82080237	4.13610835	-0.46889741

H	4.91447850	3.01554665	-0.57787156

H	6.91022621	1.15081584	-0.86619474

H	-1.91199598	3.61266078	0.12081330

Cartesian coordinates for compound MM-7.

C	5.70242971	1.08439489	-0.39604224

C	4.32378190	1.76798042	-0.31575371

C	3.20845664	1.02213551	-0.11396559

C	3.32211746	-0.50738193	0.03460398

C	4.53815854	-1.09909491	-0.03740480

C	5.79223336	-0.26046121	-0.26428982

C	4.69406494	-2.61848751	0.10543269

C	5.92947991	-3.18625459	0.02877481

C	7.20571056	-2.33280326	-0.20211694

C	7.14548945	-0.97915140	-0.33807292

C	4.21110982	3.29654270	-0.46434475

C	2.99788810	3.89648111	-0.39352260

C	1.73181150	3.04982804	-0.16445290

C	1.83032061	1.70445614	-0.03359796

C	2.05945695	-1.35871573	0.26378612

N	0.89956671	-0.79004099	0.33207480

C	-1.42381226	-0.68362604	0.62096313

N	-2.65011766	-1.10624707	0.44038632

C	-3.61246899	0.02426812	0.27851343

C	-3.03094037	1.24597455	0.38689130

S	-1.35639774	1.06315886	0.88967730

N	-0.24176991	-1.55711386	0.59466445

C	-5.11411057	-0.17635904	0.00207888

C	-6.12669383	0.90197013	0.43042510

C	-7.44813966	0.72543101	0.18715745

C	-7.93720021	-0.55351447	-0.51762436

C	-7.04612696	-1.50244696	-0.89456450

C	-5.54448227	-1.30183101	-0.61812899

F	-9.25357647	-0.72937746	-0.75995727

H	6.58422749	1.66924067	-0.55506454

H	3.83219410	-3.23210489	0.26528352

H	6.02298314	-4.24732183	0.13023020

H	8.15657634	-2.82052767	-0.25577293

H	8.04199206	-0.41689406	-0.49634432

H	5.09052140	3.88493800	-0.62348767

H	2.91959612	4.95860603	-0.49677547

H	0.77421127	3.52394113	-0.10860958

H	0.95061650	1.11649404	0.12552788

H	2.14155753	-2.42060131	0.36654173

H	-3.51710173	2.17892770	0.19158085

H	-0.35234199	-2.24616054	-0.12156858

H	-5.78689121	1.79058752	0.92011246

H	-8.15168690	1.47466284	0.48476964

H	-7.38592953	-2.39106671	-1.38424678

H	-4.84093337	-2.05105939	-0.91574567

Cartesian coordinates for compound MM-8.

C	-5.64661199	2.18872983	-0.22315507

C	-4.11525166	2.34207372	-0.14716283

C	-3.33616655	1.25545103	0.08369814

C	-3.98369703	-0.13031347	0.26962722

C	-5.33050624	-0.25507093	0.20114777

C	-6.20658610	0.96670003	-0.05856318

C	-6.01416880	-1.61650091	0.38046613

C	-7.37058461	-1.71225925	0.30607228

C	-8.26210946	-0.46894916	0.04180548

C	-7.72663552	0.77166135	-0.12721505

C	-3.46867159	3.72725669	-0.33305959

C	-2.12161474	3.86056136	-0.26594315

C	-1.23733654	2.62699940	-0.00389764

C	-1.80582769	1.40768907	0.15977861

C	-3.10371517	-1.36679132	0.53192443

N	-1.81732187	-1.24383208	0.59416520

C	0.27275494	-2.20639839	0.14720834

N	1.19837180	-3.14428980	0.12310453

C	2.42335439	-2.79598955	-0.66301813

C	2.39670754	-1.26826926	-0.80868640

S	0.73374486	-0.72870221	-0.71763942

N	3.43072672	-0.50549908	-0.95913225

N	-1.01738164	-2.36768580	0.83344147

C	2.87404463	-3.65338625	0.53427798

N	4.71334124	-1.06486999	-1.00796017

C	5.68787358	-0.10264322	-0.47342429

C	7.14208854	-0.54044261	-0.21746163

C	8.03978656	0.34660000	0.27655217

C	7.60588162	1.79242418	0.58143609

C	6.32654348	2.17786911	0.35528722

C	5.30638676	1.16990497	-0.20576930

C	8.62577951	2.80028822	1.14327855

H	-6.26432955	3.04328859	-0.40504756

H	-5.42534612	-2.49081058	0.56395221

H	-7.83358557	-2.66852971	0.43289268

H	-9.32404895	-0.58990920	-0.00884246

H	-8.36607860	1.61035656	-0.30787762

H	-4.08277375	4.58431737	-0.51517718

H	-1.67228123	4.82300048	-0.39507925

H	-0.17392136	2.73262379	0.04894849

H	-1.19137517	0.55089760	0.34190596

H	-3.55623429	-2.32757970	0.66252111

H	3.17388242	-2.84287844	-1.42420903

H	-1.47866051	-3.18483513	0.48779542

H	3.18125275	-4.61772399	0.18694902

H	2.06029860	-3.76397706	1.22011806

H	3.69373150	-3.17424785	1.02761640

H	4.73390379	-1.90026903	-0.45864744

H	7.44375013	-1.54488756	-0.42951255

H	9.05003358	0.04242957	0.45473768

H	6.02517875	3.18255704	0.56662534

H	4.29628118	1.47435365	-0.38425183

H	9.35611894	2.28048670	1.72748535

H	9.11026099	3.30603703	0.33433000

H	8.11956314	3.51460477	1.75844139

Cartesian coordinates for compound MM-9.

C	4.48615628	-2.29229177	0.01343636

C	3.08629056	-2.26741795	-0.04718819

C	2.40697670	-1.04170466	-0.05571949

C	3.12752162	0.15913522	-0.00359312

C	4.52738816	0.13426589	0.05705253

C	5.20670485	-1.09145136	0.06555522

C	5.24793908	1.33510320	0.10919879

C	6.64780691	1.31022222	0.16982512

C	7.32712218	0.08450524	0.17831058

C	6.60657051	-1.11633093	0.12618050

C	2.36573809	-3.46825661	-0.09927832

C	0.96587081	-3.44337897	-0.15991060

C	0.28655675	-2.21766273	-0.16846371

C	1.00710987	-1.01682557	-0.11636943

C	2.47056480	1.34441948	-0.01182737

N	1.17838493	1.36729013	-0.06786759

C	-0.71113341	2.46750891	-0.13144719

N	-1.46484868	1.39297063	-0.17895187

C	-2.79986529	1.65378857	-0.01129747

C	-3.10536621	2.97311814	0.13652023

S	-1.67378133	3.94354354	-0.14229043

N	0.58104433	2.44476171	-0.07529256

C	-3.73876975	0.67694849	0.01718579

C	-3.35990845	-0.66370769	-0.13456942

C	-4.33080566	-1.67386150	-0.10512507

C	-5.68056718	-1.34336051	0.07607221

C	-6.05943086	-0.00270473	0.22782660

C	-5.08853126	1.00745075	0.19838842

Cl	-6.89990593	-2.61200031	0.11304122

H	5.00483083	-3.22815171	0.01993496

H	4.72926708	2.27096523	0.10272082

H	7.19796499	2.22708694	0.20963712

H	8.39595177	0.06550828	0.22459567

H	7.12524202	-2.05219308	0.13266191

H	2.88440967	-4.40411810	-0.09275196

H	0.41571267	-4.36024577	-0.19967351

H	-0.78227219	-2.19866689	-0.21477366

H	0.48843933	-0.08096303	-0.12290596

H	3.02067122	2.26131428	0.02801677

H	-4.07141134	3.35765352	0.38908494

H	1.09507325	3.30172002	-0.03791524

H	-2.32933523	-0.91605292	-0.27292146

H	-4.04153408	-2.69748125	-0.22099269

H	-7.09000459	0.24963894	0.36617504

H	-5.37780166	2.03106997	0.31425964

Cartesian coordinates for compound MM-10

C	-4.24464227	1.91821824	0.02164426

C	-2.85540377	2.09449398	-0.03188685

C	-2.00756597	0.97875407	-0.04664554

C	-2.54896686	-0.31326180	-0.00787575

C	-3.93820530	-0.48953737	0.04565377

C	-4.78604307	0.62620250	0.06041444

C	-4.47960580	-1.78155295	0.08442365

C	-5.86884414	-1.95782902	0.13795404

C	-6.71668176	-0.84208929	0.15272180

C	-6.17528140	0.44992647	0.11395034

C	-2.31400316	3.38650962	-0.07066121

C	-0.92476497	3.56278539	-0.12419754

C	-0.07692717	2.44704569	-0.13895657

C	-0.61832750	1.15503009	-0.10017920

C	-1.72907948	-1.39221908	-0.02214426

N	-0.44670435	-1.22950056	-0.07151430

C	1.58102829	-2.04763948	-0.13382016

N	2.17355179	-0.87601382	-0.16837876

C	3.53142722	-0.94481681	0.00442042

C	4.02180022	-2.20819583	0.14303793

S	2.74487304	-3.37075657	-0.15156286

N	0.29865184	-2.21036735	-0.08451176

C	4.32090476	0.15589056	0.04608773

C	3.75493142	1.42994385	-0.09668620

C	4.57132010	2.56817661	-0.05358558

C	5.95368278	2.43235605	0.13228546

C	6.51965750	1.15830236	0.27505051

C	5.70326814	0.02006969	0.23195431

H	-4.89198510	2.77011044	0.03291474

H	-3.83226286	-2.63344498	0.07314996

H	-6.28221542	-2.94431170	0.16755355

H	-7.77739600	-0.97667995	0.19359752

H	-6.82262436	1.30181848	0.12522702

H	-2.96134599	4.23840179	-0.05939004

H	-0.51139379	4.54926791	-0.15380551

H	0.98378695	2.58163620	-0.17983468

H	0.02901565	0.30313816	-0.11144828

H	-2.14245135	-2.37870230	0.00743053

H	5.03182503	-2.45293509	0.39769964

H	-0.08767399	-3.13231772	-0.05694132

H	2.69946710	1.53364581	-0.23860514

H	4.13918611	3.54094473	-0.16259213

H	6.57701290	3.30142241	0.16519970

H	7.57512277	1.05460010	0.41696209

H	6.13540192	-0.95269826	0.34096297
